# Primary cilia formation requires the Leigh syndrome–associated mitochondrial protein NDUFAF2

**DOI:** 10.1172/JCI175560

**Published:** 2024-07-01

**Authors:** Chien-Hui Lo, Zhiquan Liu, Siyu Chen, Frank Lin, Andrew R. Berneshawi, Charles Q. Yu, Euna B. Koo, Tia J. Kowal, Ke Ning, Yang Hu, Won-Jing Wang, Y. Joyce Liao, Yang Sun

**Affiliations:** 1 Department of Ophthalmology, Stanford University School of Medicine, Palo Alto, California, USA.; 2 Department of Medicine, Stanford University, Palo Alto, California, USA.; 3 Institute of Biochemistry and Molecular Biology, College of Life Science, National Yang Ming Chiao Tung University, Taipei, Taiwan.; 4 Palo Alto Veterans Administration, Palo Alto, California, USA.; 5 Stanford Maternal and Child Health Research Institute and; 6 BioX, Stanford University School of Medicine, Palo Alto, California, USA.

**Keywords:** Cell biology, Ophthalmology, Neurodegeneration, Neurological disorders, Retinopathy

## Abstract

Mitochondria-related neurodegenerative diseases have been implicated in the disruption of primary cilia function. Mutation in an intrinsic mitochondrial complex I component *NDUFAF2* has been identified in Leigh syndrome, a severe inherited mitochondriopathy. Mutations in *ARMC9*, which encodes a basal body protein, cause Joubert syndrome, a ciliopathy with defects in the brain, kidney, and eye. Here, we report a mechanistic link between mitochondria metabolism and primary cilia signaling. We discovered that loss of NDUFAF2 caused both mitochondrial and ciliary defects in vitro and in vivo and identified NDUFAF2 as a binding partner for ARMC9. We also found that NDUFAF2 was both necessary and sufficient for cilia formation and that exogenous expression of NDUFAF2 rescued the ciliary and mitochondrial defects observed in cells from patients with known ARMC9 deficiency. NAD^+^ supplementation restored mitochondrial and ciliary dysfunction in ARMC9-deficient cells and zebrafish and ameliorated the ocular motility and motor deficits of a patient with ARMC9 deficiency. The present results provide a compelling mechanistic link, supported by evidence from human studies, between primary cilia and mitochondrial signaling. Importantly, our findings have significant implications for the development of therapeutic approaches targeting ciliopathies.

## Abstract 2

Mitochondrial metabolism is linked to primary cilia signaling and ciliopathies via the Leigh syndrome–associated mitochondrial protein NDUFAF2.

## Graphical abstract

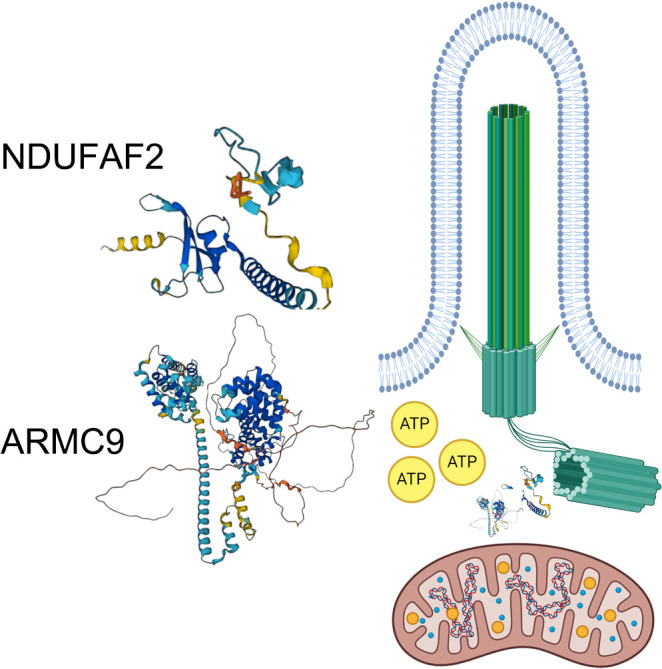

## Introduction

The primary cilium is a solitary, immobile microtubule-based structure present on the apical surface of many cell types, especially in sensory organs such as the eye and nose. The immobile sensory cilium consists of an array of bundled microtubules (axoneme) that are covered by a plasma membrane. Axonemal microtubules arise from the basal body, a specialized microtubule-nucleating center (1–5). Defects in ciliary function are associated with a spectrum of human diseases known collectively as ciliopathies, which are characterized by renal cysts, skeletal dysplasia, developmental delay, and retinal degeneration (6, 7). A rare form of syndromic ciliopathy, Joubert syndrome, is caused by mutations in genes that form the transition zone, the basal region within primary cilia that controls the trafficking of ciliary proteins. One of the Joubert syndrome proteins, ARMC9, is located at the centriole basal body and interacts with CCDC66 and TOGARAM1 to stabilize cilia membrane (8). ARMC9 loss causes photoreceptor degeneration and optic nerve and retinal coloboma (9). The precise mechanisms that link genetic mutations in the ciliary transition zone, including of ARMC9, to the systemic impairments in Joubert syndrome are unknown.

Optic nerve degeneration is also a feature of Leigh syndrome, a rare and progressive neurological disorder that affects the central nervous system and leads to early death (10, 11). Leigh syndrome is caused by mutations in mitochondrial genes, including some leading to deficiency in mitochondrial complex I (12–16), such as *NDUFAF2*, an essential assembly factor of mitochondrial complex I. *Ndufaf2*-knockout mouse fibroblasts exhibit reduced complex I activity and increased oxidative stress and mitochondrial DNA deletion (17–21). However, it is not known whether mitochondrial genes directly affect cilia function.

Mitochondria are frequently detected in the base of cilia in cells with motile cilia and photoreceptors (22, 23). A link has been proposed between ciliary and mitochondrial function, but the precise mechanism responsible for this link has not been identified. In this study, we describe a mechanism of ciliogenesis based on the interplay between mitochondrial protein NDUFAF2 and centriole protein ARMC9. We first demonstrate that ARMC9-deficient cells derived from a human patient have both ciliary and mitochondrial defects. We next show that NDUFAF2 interacts with ARMC9, restores mitochondrial defects identified in ARMC9-deficient cells, and then mediates cilia formation by removing CP110, producing ciliary vesicles, and stabilizing the transition zone. Finally, we demonstrate that NAD^+^ supplementation rescues both mitochondrial and ciliary defects in ARMC9-deficient cells and ocular motility defects in an ARMC9-deficient patient.

## Results

### Mitochondrial gene NDUFAF2 is involved in the initial steps of cilia formation.

**Figure 1:**
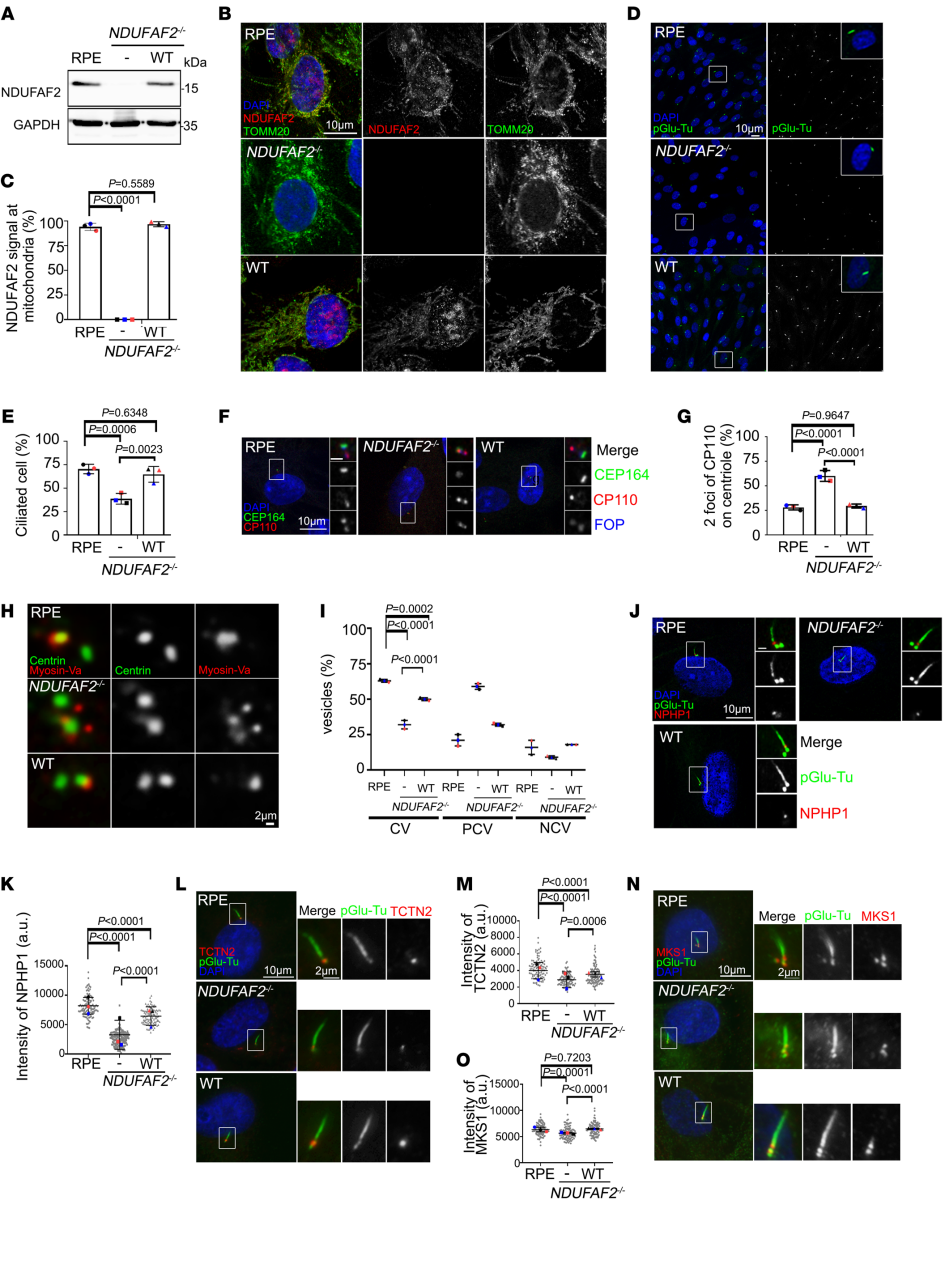
Loss of NDUFAF2 in RPE cells results in primary cilia defects. (**A**) NDUFAF2^WT^ was stably expressed in NDUFAF2^–/–^ cells. Western blot analysis was performed with antibodies against NDUFAF2 and GAPDH. (**B**) Cells stained with TOMM20 (green) and NDUFAF2 (red) antibodies. DNA stained with DAPI (blue). Scale bar: 10 μm. (**C**) NDUFAF2 staining of mitochondria in wild-type, NDUFAF2^–/–^, and NDUFAF2^WT^-re-expressing RPE1 cells. (**D**) Cells stained with polyglutamylated tubulin (green) antibodies. DNA stained with DAPI (blue). Scale bar: 10 μm. (**E**) Percentage of ciliated cells after serum starving for 2 days; >150 cells analyzed for each independent experiment. (**F**) Immunofluorescent analysis of cells serum-starved for 2 days. Cells stained with CP110 (red), CEP164 (green), and FOP (FGFR1 oncogene partner; blue) antibodies. DNA stained with DAPI (blue). Scale bars: 10 μm. (**G**) Graph shows percentage of serum-starved cells with two CP110 dots at the centrioles; >150 cells analyzed for each independent experiment. (**H**) Immunofluorescent analysis of cells serum-starved for 6 hours. Cells were stained with myosin-Va (red) and centrin (green) antibodies. DNA stained with DAPI (blue). Scale bar: 2 μm. (**I**) Percentage of cells with ciliary vesicles demonstrated by an antibody against myosin-Va after serum starving for 6 hours (CV, ciliary vesicle; NCV, no ciliary vesicle; PCV, preciliary vesicle); >150 cells analyzed for each independent experiment. (**J**) Immunostaining of cells serum-starved for 2 days. Scale bars: 10 μm. (**K**) Quantification of NPHP1 signal intensity at the centrioles; >100 cells analyzed for each independent experiment. (**L**) Immunofluorescent analysis of cells serum-starved for 2 days. Cells stained with TCTN2 (red) and pGlu-Tu (green) antibodies. DNA stained with DAPI (blue). Scale bars: 10 μm, 2 μm. (**M**) Quantification of TCTN2 signal intensity at the centrioles; >50 cells analyzed for each independent experiment. (**N**) Immunofluorescent analysis of cells serum-starved for 2 days. Cells stained with MKS1 (red) and pGlu-Tu (green) antibodies. DNA stained with DAPI (blue). Scale bars: 10 μm, 2 μm. (**O**) Quantification of MKS1 signal intensity at the centrioles; >50 cells analyzed for each independent experiment. Bars represent mean ± SD, *n* = 3. Exact *P* values are indicated. ANOVA followed by Tukey-Kramer multiple-comparison test.

Mutations of mitochondrial genes have been reported to affect ciliogenesis and cilia-dependent processes in zebrafish, human fibroblasts, and *Tetrahymena thermophila*, but the mechanism is still unclear (22, 24–26). Here, we identified a family with retinal and optic nerve degeneration and a previously unreported *NDUFAF2* mutation. The proband was a 28-year-old woman who presented with retinal degeneration and optic nerve atrophy, with hyperpigmentary changes diffusely in retinal pigment epithelial (RPE) cells (Supplemental Figure 1; supplemental material available online with this article; https://doi.org/10.1172/JCI175560DS1). To understand how a defective mitochondrial protein contributes to ciliogenesis, we created stable clonal RPE cell lines, using CRISPR/Cas9–induced technology, to knock out the *NDUFAF2* gene. We re-expressed wild type (NDUFAF2^WT^) in the NDUFAF2-knockout cells, where it continued to be stably expressed (Figure 1, A–D). Genotyping showed a clone of multiple base deletions in both alleles, which were predicted to produce severely truncated products consisting of only 38 amino acids in both types of knockout clone cells (Supplemental Figure 2A). We confirmed a mitochondrial defect by determining that the oxygen consumption rate (OCR), complex I activity, and NAD^+^/NADH ratio were markedly decreased in NDUFAF2^–/–^ cells (Supplemental Figure 2, B–D). However, no significant mitochondrial crista defects were noted by transmission electron microscopy (TEM) in NDUFAF2^–/–^ cells (Supplemental Figure 2E). We then confirmed the signal of NDUFAF2^–/–^ in mitochondria. NDUFAF2^–/–^, NDUFAF2^WT^-re-expressing, and RPE cells were stained for NDUFAF2 and TOMM20. The results confirmed that NDUFAF2^–/–^ cells did not express any NDUFAF2 protein, indicating successful knockout (Figure 1, B and C).

Based on the clinical observation of photoreceptor degeneration, we hypothesized that cilia function is affected in the NDUFAF2-deficient cells. Indeed, these NDUFAF2-deficient cells exhibited cilia formation defects upon serum starvation (Figure 1, D and E). To determine how NDUFAF2 contributed to ciliogenesis, we analyzed the key steps during ciliogenesis, including removal of CP110 from the mother centrioles and the docking of membrane vesicles to the mother centrioles. We found that most of the NDUFAF2^–/–^ cells continued to show two CP110 foci at the centrioles, indicating that the removal of CP110 was regulated by NDUFAF2 (Figure 1, F and G). CEP97 and CEP290 have both been demonstrated to interact with CP110 and inhibit ciliogenesis (27, 28). Here, we found that most of the NDUFAF2^–/–^ cells continued to show two CEP97 foci at the centrioles (Supplemental Figure 3, A and B), that CEP290 was still localized at the pericentriolar material (PCM) (Supplemental Figure 3, C and D), and that there were no significant differences among the protein levels of CP110, CEP97, and CEP290 (Supplemental Figure 3E). Furthermore, we examined the docking of ciliary membrane vesicles to the centrioles by immunostaining and transmission electron microscopy; we found that NDUFAF2^–/–^ cells contained fewer ciliary vesicles than wild-type RPE and NDUFAF2^WT^-re-expressing cells, indicating that NDUFAF2 affected ciliary vesicle docking (Figure 1, H and I, and Supplemental Figure 3F). Moreover, NDUFAF2 did not affect the migration of intraflagellar transport 88 (IFT88) into the cilium (Supplemental Figure 3, G and H). Further, we examined several transition zone proteins in the cilia, including NPHP1, which is mutated in nephronophthisis (OMIM 607100), TCTN2, MKS1, and MKS3, using immunostaining and TEM; we found that NPHP1, TCTN2, and MKS1 were markedly decreased in NDUFAF2^–/–^ RPE cells, indicating that NDUFAF2 regulates the establishment of the transition zone (Figure 1, J–O, and Supplemental Figure 3, I–L). Taken together, these results show that NDUFAF2 is involved in the initial steps of cilia formation, including the docking of membrane vesicles, removal of CP110, and establishment of the transition zone.

### Supplemental ATP promotes stabilization of the transition zone.

Adenosine triphosphate (ATP) has well-recognized roles in energy transfer and signal transduction, but the specific role of ATP in stabilizing the formation of transition zones may depend on the subcellular milieu (29–33). Here, we hypothesize that ATP indirectly affects transition zone stability by providing energy for the molecular machinery involved in its formation and maintenance; ATP hydrolysis fuels the activities of motor proteins such as kinesins and dyneins, which are involved in transporting cellular components and organizing cellular structures (29). To test our hypothesis, we applied supplemental ATP to RPE, NDUFAF2^–/–^, and NDUFAF2^WT^-re-expressing cells and assessed the transition zone proteins. The intensity of NPHP1 increased in NDUFAF2^–/–^ cells when additional ATP was provided, supporting that ATP can stabilize protein complexes or promote the assembly of molecular structures critical for transition zone formation (Supplemental Figure 4).

### NDUFAF2 mutants exhibit a ciliopathy phenotype in vivo.

**Figure 2:**
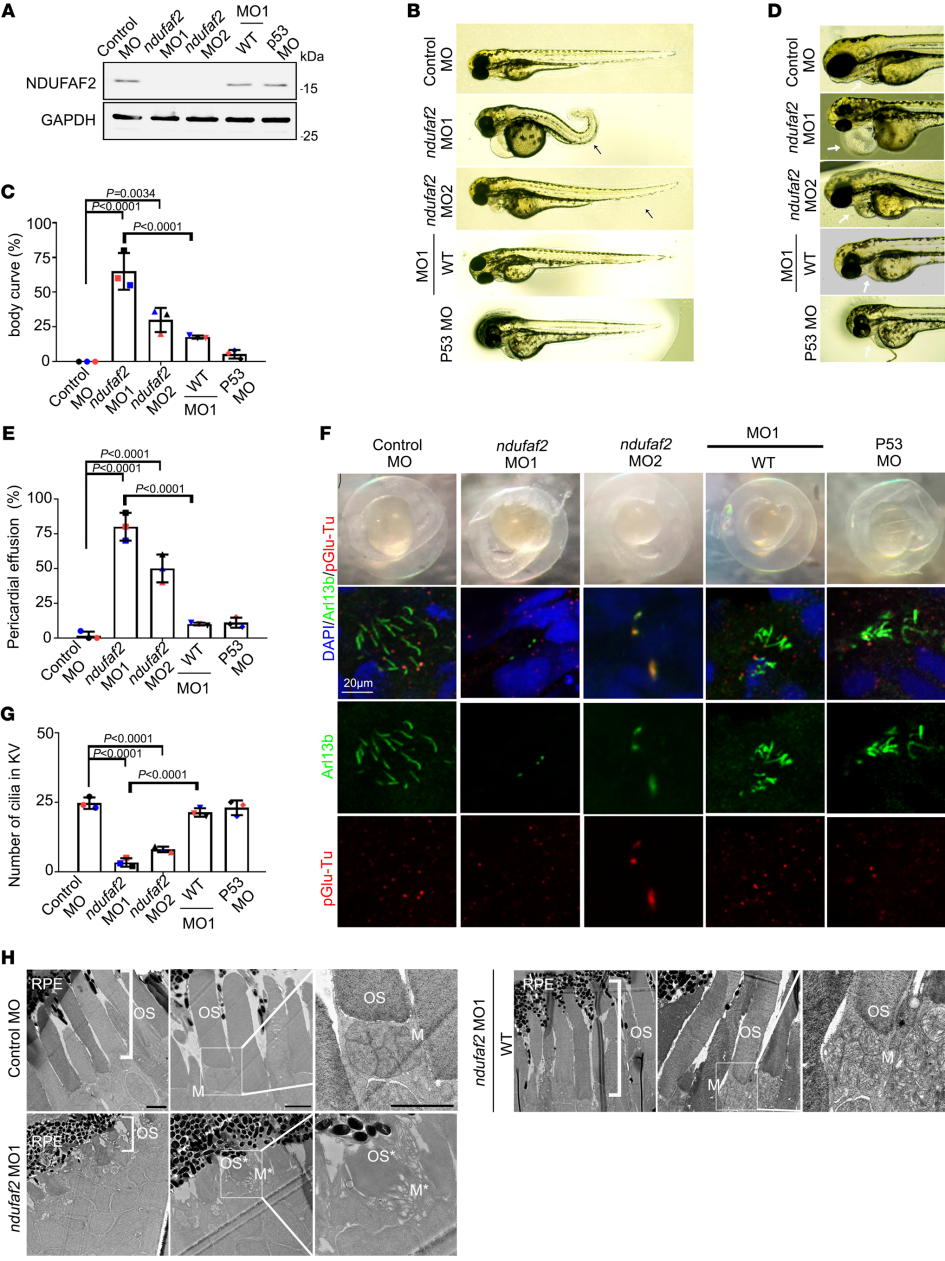
Zebrafish *ndufaf2* morpholino–injected mutants exhibit a ciliopathy phenotype. (**A**) Protein levels of control morpholino (MO)–injected, *ndufaf2* morpholino–injected, and *p53* morpholino–injected embryos and *ndufaf2* morpholino–injected embryos re-expressing *NDUFAF2^WT^*. Western blot analysis was performed with antibodies against NDUFAF2 and GAPDH. (**B**) Transmitted light images of body shape in control MO–, *ndufaf2* morpholino–, and *p53* morpholino–injected zebrafish larvae and *ndufaf2* morpholino–injected zebrafish larvae re-expressing *NDUFAF2^WT^*, at 5 days post-fertilization (dpf). (**C**) Quantification of body curve in control MO–, *ndufaf2* morpholino–, and *p53* morpholino–injected zebrafish larvae and *ndufaf2* morpholino–injected zebrafish larvae re-expressing *NDUFAF2^WT^*, at 5 dpf; >30 larvae analyzed for each independent experiment. (**D**) Transmitted light images of heart in control MO–, *ndufaf2* morpholino–, and *p53* morpholino–injected zebrafish larvae and *ndufaf2* morpholino–injected zebrafish larvae re-expressing *NDUFAF2^WT^*, at 5 dpf. Arrows indicate the heart. (**E**) Quantification of heart failure in control MO–, *ndufaf2* morpholino–, and *p53* morpholino–injected zebrafish larvae and *ndufaf2* morpholino–injected zebrafish larvae re-expressing *NDUFAF2^WT^*, at 5 dpf; >30 larvae analyzed for each independent experiment. (**F**) Confocal images of Kupffer’s vesicle (KV) cells labeled with cilia markers, Arl13b (green) and pGlu-Tu (red), in control MO–, *ndufaf2* morpholino–, and *p53* morpholino–injected zebrafish larvae and *ndufaf2* morpholino–injected zebrafish larvae re-expressing *NDUFAF2^WT^*, at 8 somite stage. Scale bar: 20 μm. (**G**) Quantification of KV cilia number in control MO–, *ndufaf2* morpholino–, and *p53* morpholino–injected zebrafish larvae and *ndufaf2* morpholino–injected zebrafish larvae re-expressing *NDUFAF2^WT^*; >30 larvae analyzed for each independent experiment. (**H**) TEM images of *ndufaf2*-mutant zebrafish showing shortened and disorganized photoreceptor outer segments at 5 dpf. Representative images of control MO– and *ndufaf2* morpholino–injected zebrafish larvae and *ndufaf2* morpholino–injected zebrafish larvae re-expressing *NDUFAF2^WT^*, at 5 dpf. Boxes show areas of enlarged mitochondria. M,  mitochondria; M*, swollen mitochondria; OS*, shorter  outer segment of photoreceptor. Scale bars: 2 μm. The bars in each graph represent mean ± SD. Exact *P* values are indicated. ANOVA followed by Tukey-Kramer multiple-comparison test.

Zebrafish model systems are commonly used to study human ciliopathies owing to the highly conserved ciliary genes in zebrafish and humans. Here, we knocked down *ndufaf2* in zebrafish using morpholinos and assessed these morphants by coinjection of *ndufaf2^WT^* mRNA (Figure 2A). Zebrafish with *ndufaf2* knocked down displayed multiple ciliopathic phenotypes, including abnormal dorsal body curvature, defective optic fissure closure, and cardiac edema (Figure 2, B–E, Supplemental Figure 5, A and B, and Supplemental Videos 1–4), but not otic vesicle defects (Supplemental Figure 5, C and D). Furthermore, the numbers of cilia in Kupffer’s vesicle were markedly decreased in embryos in which *ndufaf2* was knocked down; these defects could be rescued by coinjection of *ndufaf2^WT^* mRNA (Figure 2, F and G). NDUFAF2 mutants also exhibited photoreceptor dysfunction and degeneration in vivo, including a shorter than normal photoreceptor outer segment and reduced numbers of retinal ganglion cells compared with wild type (Figure 2H and Supplemental Figure 6, A–G). To assess the functional impact of *ndufaf2* loss, we used calcium indicators for photoreceptor measurements. Different neurodegenerative disorders may result in disrupted calcium regulation, potentially causing photoreceptor loss and retinal ganglion cell degeneration (34–36). Here, we used fluo-4 staining to measure Ca^2+^ signaling in live images of larvae’s eyes (37). Knockdown of *ndufaf2* showed markedly decreased fluo-4 signals in both photoreceptors and retinal ganglion cells (RGCs) compared with wild type (Supplemental Figure 6H). The observed defects in the zebrafish model closely resemble those seen in the human patient, supporting an essential role of NDUFAF2 in photoreceptors and optic nerve. Taken together, these results suggest that NDUFAF2 is necessary for cilia formation.

### Centrosome protein ARMC9 interacts with NDUFAF2.

**Figure 3:**
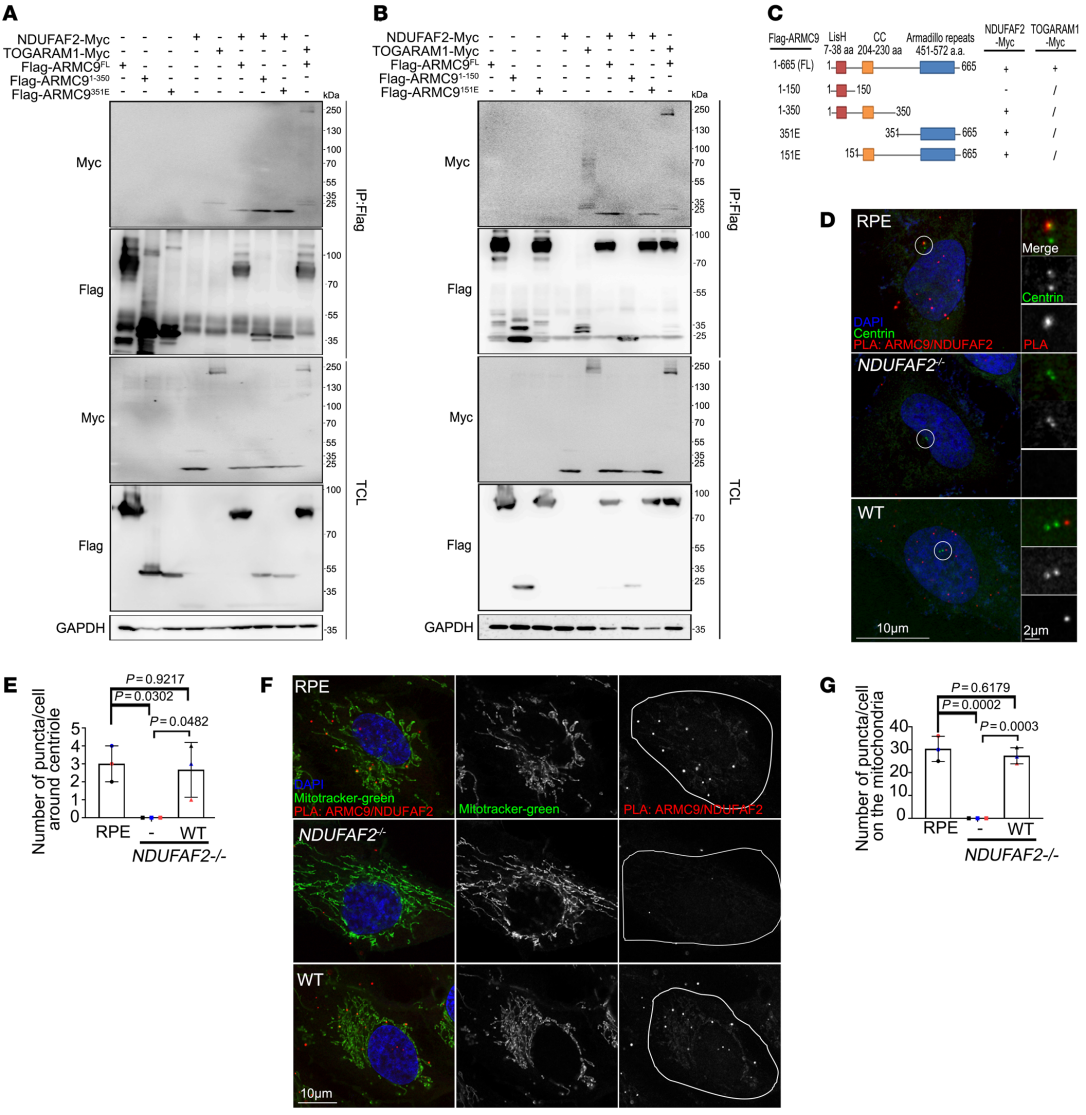
ARMC9 interacts with NDUFAF2 via its C-terminal region, and their interaction is required for localization of mitochondria and centrosome. (**A** and **B**) 293T cells transfected with various expression constructs analyzed by immunoprecipitation followed by Western blots with indicated antibodies. (**C**) Analysis of 293T cells transfected with various expression constructs by immunoprecipitation followed by Western blots. Schematic diagram showing various ARMC9 mutants tagged with FLAG. The ability of each construct to interact with NDUFAF2 is also indicated. CC, mean coiled-coil domain; LisH, mean predicted lissencephaly type 1–like homology motif. (**D**) Cells were incubated with a centriole marker (centrin, green) and with anti-ARMC9 and anti-NDUFAF2 antibodies and then probed using PLA Minus anti-ARMC9 and Plus anti-NDUFAF2 (red). Scale bars: 10 μm, 2 μm. (**E**) Quantification of puncta around centrioles in wild-type, NDUFAF2^–/–^, and NDUFAF2^WT^-re-expressing RPE1 cells. (**F**) Cells were incubated with a mitochondrial marker (green) and with anti-ARMC9 and anti-NDUFAF2 antibodies and then probed using PLA (red). Scale bar: 10 μm. (**G**) Quantification of puncta on mitochondria in wild-type, NDUFAF2^–/–^, and NDUFAF2^WT^-re-expressing RPE1 cells. The bars in each graph represent mean ± SD. Exact *P* values are indicated. ANOVA followed by Tukey-Kramer multiple-comparison test.

To further assess NDUFAF2’s involvement in ciliary biology, we found that a ciliary protein, armadillo repeat containing 9 (ARMC9), interacts with NDUFAF2 via its coiled-coil region and armadillo repeat (Figure 3, A–C). Since ARMC9 has previously been recognized as a basal body protein, we assessed the in vitro interaction between ARMC9 and NDUFAF2 using proximity ligation assay (PLA). We found that PLA-positive dots, which reflect the ARMC9-NDUFAF2 interaction, were decreased at the basal bodies of centrioles in NDUFAF2^–/–^ cells, indicating that NDUFAF2 may localize at the basal body of centrioles (Figure 3, D and E). Moreover, PLA-positive dots were depleted on the mitochondria in NDUFAF2^–/–^ cells, indicating that the interaction is crucial to the localization of mitochondria (Figure 3, F and G).

### NDUFAF2 rescues the mitochondrial defect and cilia formation in a patient with Joubert syndrome.

**Figure 4:**
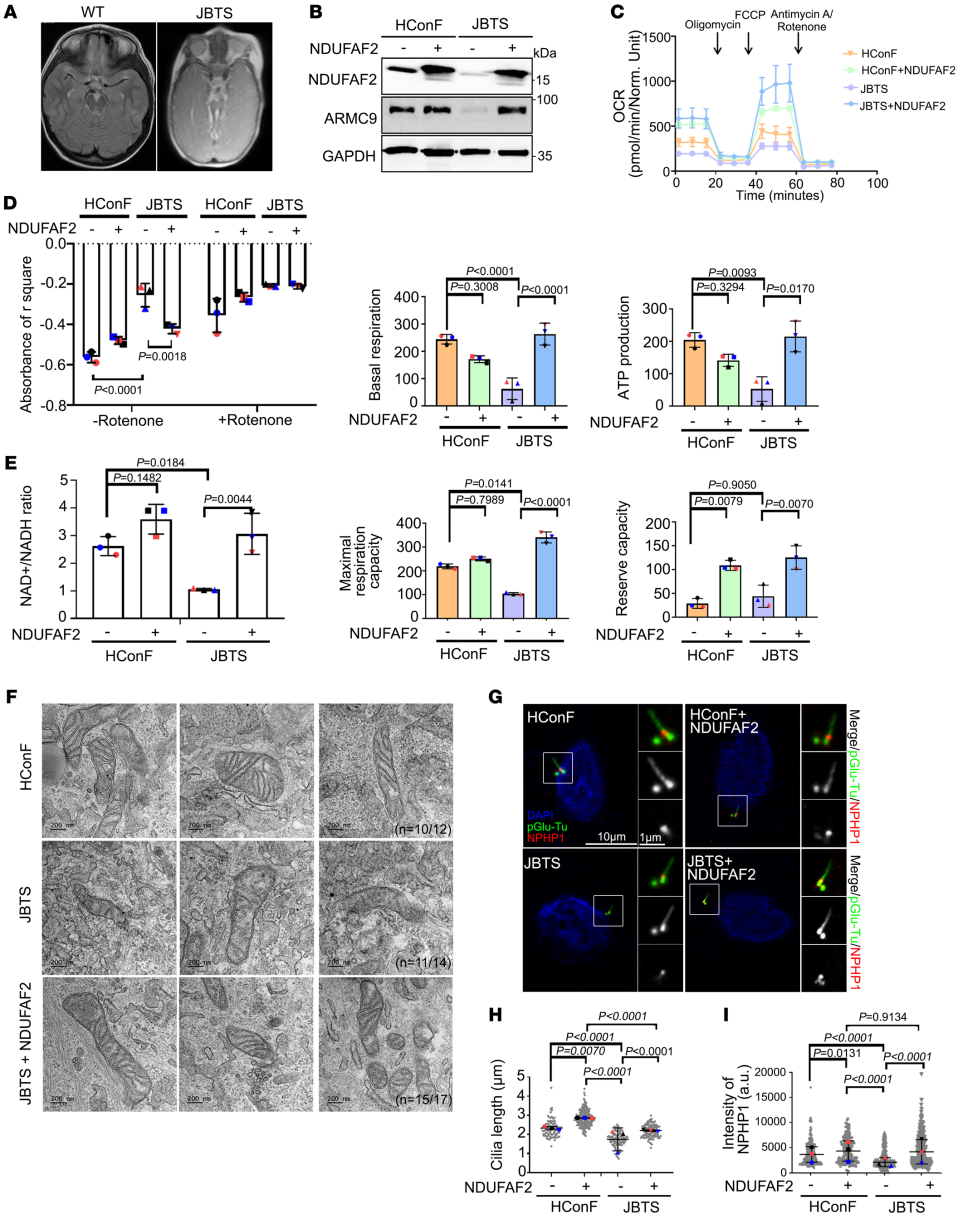
NDUFAF2 rescues the mitochondrial defects in Joubert syndrome patient–derived cells. (**A**) Brain MRI T1 sequence of a JB patient demonstrating molar tooth sign (MTS) in midbrain. (**B**) NDUFAF2 is stably expressed in human conjunctival fibroblasts (HConF) and Joubert syndrome patient–derived cells (JBTS), with compound heterozygous mutation in ARMC9. Western blot analysis performed with antibodies against NDUFAF2, ARMC9, and GAPDH. (**C**) Oxygen consumption rate (OCR) of HConF cells and JBTS cells measured by Seahorse Analyzer. (**D**) Analysis of mitochondrial complex I activity in HConF cells, JBTS cells, and both cell lines overexpressing NDUFAF2. (**E**) Analysis of the NAD^+^/NADH ratio in HConF cells and JBTS cells. (**F**) TEM images of HConF cells, JBTS cells, and JBTS cells overexpressing NDUFAF2. Eleven JBTS cells display tube-like cristae in stack and onion shapes. The total number of mitochondria in HConF cells, JBTS cells, and JBTS cells overexpressing NDUFAF2 is 12, 14, and 17, respectively. Ten of 12 mitochondria are normal in HConF cells; 11 of 14 mitochondria display onion shapes in JBTS cells; 15 of 17 mitochondria are normal in JBTS cells overexpressing NDUFAF2. Scale bars: 200 nm. (**G**) Immunostaining of cells serum-starved for 2 days. Scale bars: 10 μm, 1 μm. (**H**) Quantification of cilia length in HConF cells, JBTS cells, and both cell lines overexpressing NDUFAF2. (**I**) Quantification of NPHP1 signal intensity at the centrioles; >50 cells analyzed for each independent experiment. The bars in each graph represent mean ± SD. Exact *P* values are indicated. ANOVA followed by Tukey-Kramer multiple-comparison test.

Building on the observation that NDUFAF2 deficiency results in ciliary abnormalities, we investigated a Joubert syndrome family with a known mutation within ARMC9. The proband patient was a 9-year-old boy with molar tooth sign in the midbrain, who developed optic nerve colobomas, ptosis, and retinal degeneration (Figure 4A and Supplemental Figure 7) (38). The child carried *ARMC9* mutations (case number UW349-3, c.1474+1G>C and c.1027C>T) (8, 9). We generated a patient-derived fibroblast line (JBTS cells) and verified a previously noted ciliary defect (Figure 4, G–I, and Supplemental Figure 9E) (8).

We found that NDUFAF2 protein levels and the interaction between NDUFAF2 and ARMC9 were reduced in JBTS cells compared with wild-type human conjunctival fibroblast (HConF) cells (Figure 4B and Supplemental Figure 8). We also found that the patient-derived fibroblasts exhibited a mitochondria-defective phenotype: the OCR, complex I activity, and NAD^+^/NADH ratio were dramatically reduced in JBTS cells as compared with the HConF cells. Importantly, overexpression of wild-type NDUFAF2 in JBTS cells largely rescued the mitochondrial OCR defects (Figure 4, C–E).

To assess mitochondrial structure, we used electron microscopy to compare mitochondrial cristae in NDUFAF2-transfected cells and untreated patient-derived (JBTS) cells. Based on the OCR defects, we hypothesized that the structure of mitochondrial cristae in JBTS cells, control (HConF) cells, and NDUFAF2-overexpressing JBTS cells would differ. We found that there were more onion-shaped cristae and fewer cristae overall in JBTS patient-derived cells than in the HConF cells, and that NDUFAF2 overexpression rescued this phenotype (Figure 4F). Moreover, the JBTS patient-derived cells showed a greater number of filamentous mitochondria and higher oxidative stress levels than control cells, which could be rescued by NDUFAF2 overexpression (Supplemental Figure 9, A–D). Therefore, we concluded that ARMC9-deficient cells derived from a patient with Joubert syndrome exhibit mitochondrial defects, which can be rescued by NDUFAF2 expression.

To evaluate the impact of NDUFAF2 on cilia formation, we assessed whether NDUFAF2 expression would also rescue the ciliary defects in JBTS cells. The percentage of ciliated cells was not significantly different in JBTS cells and control cells (HConF), but cilia were shorter in patient-derived JBTS cells (Figure 4, G and H, and Supplemental Figure 9E). Because previous reports have noted that mutations in JBTS cells affected the ciliary transition zone (39), we assessed the localization of NPHP1 in JBTS patient-derived cells. NPHP1 staining was less intense in JBTS cells than in control cells; in NDUFAF2-overexpressing JBTS cells, NPHP1 continued to localize to the transition zone (Figure 4, G and I). Furthermore, overexpression of ARMC9 was not sufficient for cilia formation and mitochondrial activities (Supplemental Figure 10), but ARMC9 was required for cilia formation (Supplemental Figure 11). Therefore, ARMC9 interacts with NDUFAF2, which is involved in ciliary transition zone formation.

### NAD^+^ supplementation rescues the mitochondrial and cilia defects in vivo and in JBTS cells.

**Figure 5:**
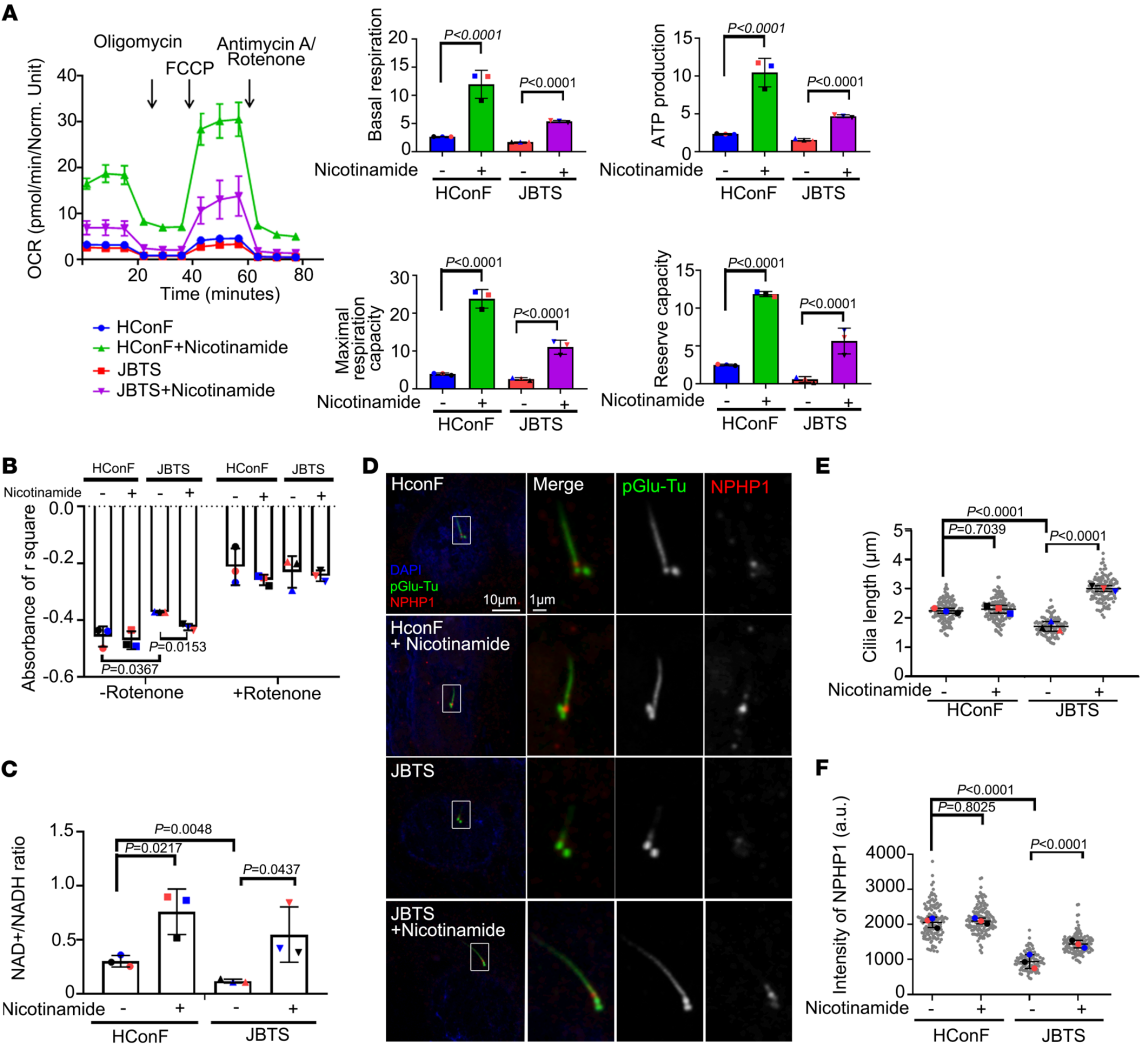
NAD^+^ supplementation rescues defective ciliogenesis in JBTS patient-derived cells. (**A**) OCR measured by Seahorse Analyzer of HConF cells and JBTS cells with or without nicotinamide treatment. (**B**) Analysis of mitochondrial complex I activity in HConF cells, JBTS cells, and both cell lines overexpressing NDUFAF2. (**C**) Analysis of the NAD^+^/NADH ratio in HConF cells and JBTS cells. (**D**) Immunostaining of cells serum-starved for 2 days. Scale bars: 10 μm, 1 μm. (**E**) Quantification of cilia length in HConF cells and JBTS cells treated with nicotinamide; >50 cells analyzed for each independent experiment. (**F**) Quantification of NPHP1 signal intensity at the centrioles; >100 cells analyzed for each independent experiment. The bars in each graph represent mean ± SD. Exact *P* values are indicated. ANOVA followed by Tukey-Kramer multiple-comparison test.

Mitochondrial complex I plays an important role in releasing reactive oxygen species and oxidizing NADH (40, 41). We hypothesized that metabolic supplementation to increase mitochondrial metabolic pathways would rescue mitochondrial and ciliary defects in JBTS cells. To test this idea, we provided nicotinamide, a precursor form of vitamin B_3_ containing NAD^+^, as a substrate of complex I (42). Treatment of JBTS cells with nicotinamide elevated levels of OCR, increased complex I activity, and reduced NAD^+^/NADH ratio in comparison with untreated JBTS cells (Figure 5, A–C). Consistent with our hypothesis, nicotinamide also reversed the deficiencies in cilia length and NPHP1 levels found in untreated JBTS cells (Figure 5, D–F).

In a separate approach, we examined the effect of nicotinamide in *armc9*-deficient zebrafish. Zebrafish larvae with *armc9* knocked down displayed a thinner photoreceptor layer than wild type, but body curve was unaffected (Supplemental Figure 12, A–D) (9). Larvae with morpholino-mediated knockdown of *armc9* showed a thinned rod outer segment; this defect can be rescued by coinjection of *ndufaf2* mRNA. Separately, treatment of *armc9*-deficient zebrafish larvae with solubilized nicotinamide restored these rod outer segment defects (Supplemental Figure 12E). Thus, NAD^+^ supplementation successfully remedied the mitochondrial defects both in JBTS cells and in zebrafish larvae lacking ARMC9. Taken together, the results of NAD^+^ supplementation show that the mitochondrial metabolic pathway contributes to the early steps of ciliogenesis by stabilizing transition zone proteins. Through these mechanisms, NAD^+^ supplementation can reverse the mitochondrial and ciliary defects in JBTS cells and consequently provide a potential therapy for patients with ciliopathies.

### NAD^+^ supplementation improves ocular saccades in a patient with Joubert syndrome.

**Figure 6:**
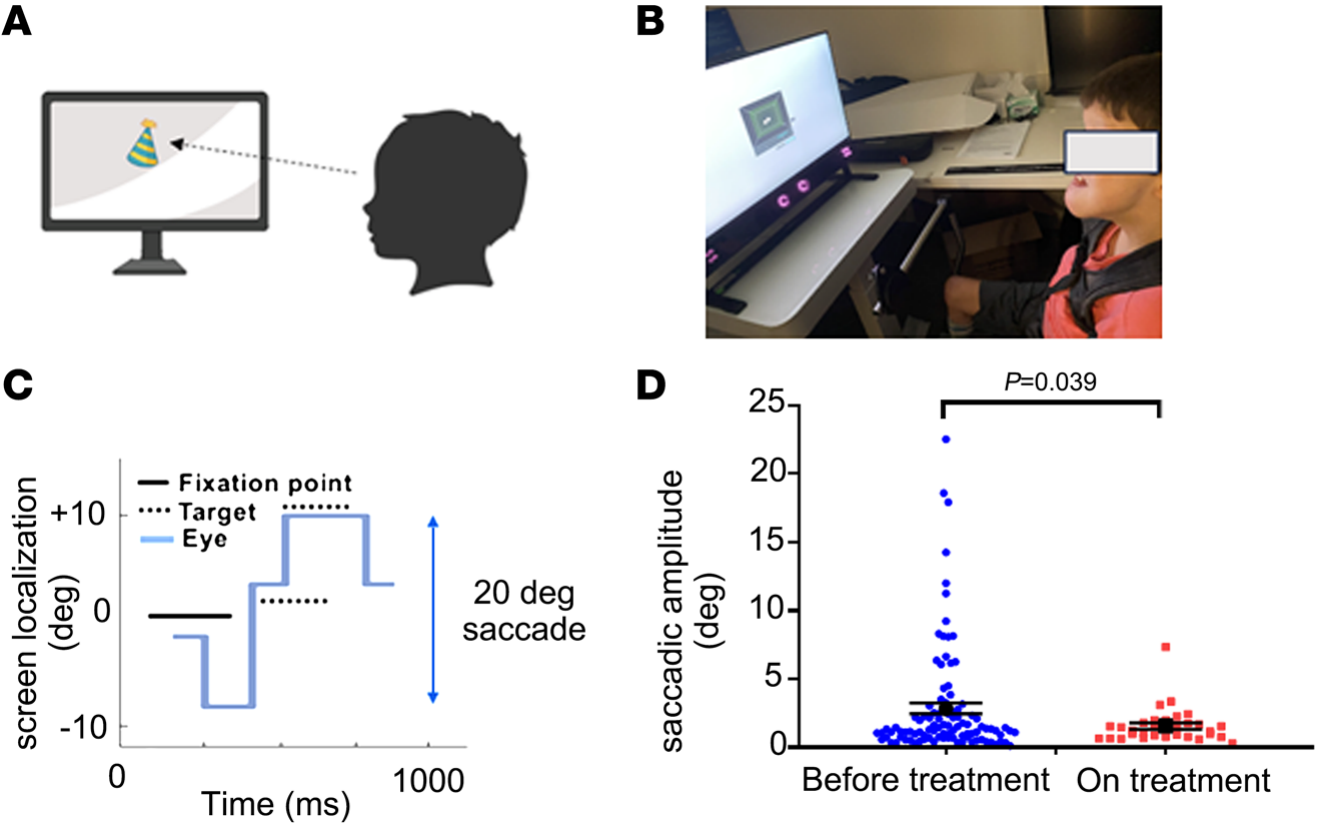
Improvement in saccadic amplitude in Joubert syndrome patient upon nicotinamide supplementation. (**A**) Schematic of eye tracking design. (**B**) Joubert syndrome patient undergoing examination. (**C**) Representative image of target, fixation point, and eye positions for eye tracking exam. (**D**) Saccadic amplitude and SEM for the last 2 sessions (101 trials) before and on treatment with nicotinamide (oral 250 mg daily for 2 months) (8,707 saccades, before-treatment group, vs. 3,359 saccades, on-treatment group). Exact *P* values are indicated. One-tailed Student’s *t* test.

Patients with Joubert syndrome demonstrate congenital ocular motor apraxia with difficulty initiating ocular saccades and central apneic suppression that shortens life expectancy. Based on the in vitro and in vivo evidence supporting the benefits of nicotinamide in ARMC9 deficiency, the pediatric team caring for patient JB recommended treatment with low-dosage nicotinamide. Because previous clinical studies showed that daily oral supplementation with 250 mg of nicotinamide for 2 months is safe (43), the patient received 250 mg of nicotinamide. Ocular saccadic movements measured by the Tobii Pro Spectrum system were used as endpoints for ocular motility and motor function. Treatment with low-dose NAD^+^ supplementation markedly improved the patient’s motor skills according to the parents’ estimation, and the results of objective eye movement studies confirmed this impression (Figure 6).

## Discussion

Our data demonstrate that an abnormality of a nuclear-encoded, intrinsic mitochondrial protein, NDUFAF2, impairs the function of a centriole protein, ARMC9, during ciliogenesis. We also report that normal NDUFAF2 function during ciliogenesis depends on a tightly regulated ratio of NAD^+^/NADH. Specifically, we find that the mitochondrial protein NDUFAF2 is involved in membrane vesicle docking, CP110 removal, and transition zone stabilization during cilia formation. Importantly, we show that a patient with Joubert syndrome due to ARMC9 mutation had a decreased level of NDUFAF2 protein, deficient mitochondrial activity, and increased oxidative stress. Overexpression of NDUFAF2 rescued the mitochondrial defect and cilia formation in JBTS fibroblast cells and *armc9*-deficient zebrafish larvae. These findings establish a functional role of mitochondria in supporting the initiation of ciliogenesis and identify a human-disease-relevant linkage between mitochondria and cilia.

In this study, we propose that ARMC9 interacts with NDUFAF2 to carry out its functions in the transition zone. Cellular functions such as apoptosis regulation, ER stress response, and phospholipid synthesis are coordinated by relatively stable contacts between the ER and mitochondria (44–46). Additionally, recent studies have demonstrated that the transition zone acts as a lipid gate and that it plays a conserved role in maintaining the phosphatidylinositol composition of ciliary membranes (47, 48). Our hypothesis is that overexpression of NDUFAF2 will provide a functional link between mitochondria and the ER that will facilitate the coordination of cellular biological functions, including phospholipid synthesis, and that NDUFAF2 expression will then stabilize transition zone formation. However, the mechanisms by which ARMC9 and NDUFAF2 coordinately influence ciliary composition and function are presently unclear.

The mechanism by which NDUFAF2 interacts with ARMC9 is also not fully elucidated. One possibility is that NDUFAF2 is located on the outer membrane of mitochondria, allowing it to interact with ARMC9. Alternatively, NDUFAF2 could be released from mitochondria and interact with ARMC9 after diffusing into the cytosol. This interaction may involve the intrinsically disordered protein regions (IDRs), which we hypothesize to play an important role in primary cilia assembly. We base this suggestion on our comparison of centriole protein ARMC9 and mitochondrial protein NDUFAF2 by sequence prediction (Predictor of Natural Disordered Regions, PONDR), which showed that both contain an IDR at the C-terminal region. IDRs are associated with several human diseases and play important roles in diverse cellular processes, including transcription, translation, cell signaling, protein interaction, and gene regulation (49, 50). Overall, IDRs may play diverse essential roles in cilia formation, including regulation of protein-protein interactions, molecular flexibility such as intraflagellar transport (IFT), modulation of signaling pathways, and scaffolding of protein complexes. Understanding the functions of IDRs in cilia formation is crucial for elucidating the molecular mechanisms underlying ciliary biology and diseases.

We found the increase of ARMC9 after stable transfection with NDUFAF2. Furthermore, autophagy is a cellular process responsible for degrading and recycling damaged organelles and proteins, thereby maintaining cellular homeostasis (51–53). Dysregulation of autophagy has been implicated in ciliopathies (54–57). Here, we hypothesize that this dysregulation may contribute to the pathogenesis of Joubert syndrome by impairing cellular quality control mechanisms and leading to the accumulation of dysfunctional cellular components. However, the exact mechanisms underlying the relationship between NDUFAF2 overexpression and autophagy inhibition in Joubert syndrome require further investigation.

Several over-the-counter supplements increase NAD levels, including niacin, nicotinamide riboside (NR), and nicotinamide mononucleotide, and vitamin B_3_ supplementation has been in use for several decades. Because no severe side effects are reported, these interventions are considered relatively safe for human use (58). Short-term experiments showed that high doses of nicotinamide are toxic to the liver (59). Niacin may cause headaches, flushing of the skin, and dizziness in high doses (60). Long-term human trials showed no effect of nicotinamide on the development of diabetes, but animal data contradict this conclusion (59), and high doses of NR produce insulin resistance and white adipose tissue dysfunction in mice (61). Overall, while NAD^+^ supplementation holds promise as a treatment for various health conditions, including aging-related diseases (62, 63), further research is needed on its long-term implications and safety profile. Ongoing studies will determine the effects of sustained treatment in specific patient populations, including those with chronic schizophrenia, Parkinson’s disease, chronic fatigue syndrome, and type 1 diabetes (58, 64–67).

The present results also show that ATP plays a significant role in stabilizing the formation of the ciliary transition zone (48). Possible mechanisms by which ATP may act include providing energy for intraflagellar transport (68, 69), regulating protein complex assembly (70, 71), maintaining ion gradients (72), and modulating signaling pathways (73).

The findings provide evidence supporting mitochondria’s functional role in ciliogenesis and reveal a link between mitochondria and cilia. Mitochondria play a crucial role in numerous cellular functions, including energy production, metabolism regulation, and cell signaling. Loss or impairment of mitochondrial activity can have significant consequences for various cellular processes. Further research is required to determine exactly how mitochondria play an important role in ciliogenesis.

In conclusion, by using evidence from a patient with a ciliopathy to establish a mechanistic connection between mitochondrial signaling and primary cilia, this research offers a therapeutic perspective for patients with ciliopathies.

## Methods

### Sex as a biological variable.

Our study examined a male patient; sex was not considered as a biological variable.

### Cell culture and reagents.

HEK293T and HEK293FT cells were cultured in DMEM supplemented with 10% FBS and 1% penicillin-streptomycin. The human telomerase-immortalized RPE cells (hTERT-RPE1 or RPE1) were cultured in DMEM/F-12 (1:1) medium supplemented with 10% FBS and 1% penicillin-streptomycin. The human conjunctival fibroblasts (HConF, ScienCell Research Laboratories, 6570) from healthy human conjunctiva and conjunctiva cells from a patient with JBTS were cultured in DMEM/F-12 (1:1) medium supplemented with 10% FBS, Fibroblast Growth Supplement (FGS, ScienCell Research Laboratories, 2352), and 1% penicillin-streptomycin.

### Plasmids.

The human NDUFAF2, ARMC9, and TOGARAM1 cDNA was obtained from Origene (RC207387, RC209499, and RC222959). To generate the epitope-tagged ARMC9, fragments were amplified by PCR and cloned into pcDNA_3_-FH. The pcDNA_3_-FH vector was derived from pcDNA_3_ (Thermo Fisher Scientific) but contained sequences for FLAG and HA epitope tag between the HindIII and BamHI cloning sites. Thus, the expressing proteins expressed FLAG and HA epitope tag at the N-terminus. Various ARMC9 mutants were cloned to pcDNA_3_-FH vector and used to analyze ARMC9-NDUFAF2 interaction (Figure 4, A and B, and Supplemental Figure 3). The NDUFAF2 and ARMC9 fragments were also subcloned into pCS vector from Addgene (plasmid 12158) so that the constructs could be stably expressed in RPE1 cells, human conjunctival fibroblasts, and JBTS patient-derived fibroblasts.

### Primary antibodies.

Primary antibodies were obtained from the following sources and used according to the manufacturers’ instructions: rabbit anti-NDUFAF2 (Western blot [WB] 1:500; HPA054776, MilliporeSigma), mouse IgG2a anti-CEP164 (immunofluorescence [IF] 1:1,000; sc-515403, Santa Cruz Biotechnology), rabbit anti-CP110 (WB 1:1,000, IF 1:1,000; 12780-1-AP, Proteintech), mouse IgG2b anti-FOP (IF 1:1,000; H00011116-M01, Abnova), anti–polyglutamylated tubulin (pGlu-Tu) (IF 1:3,000; 901501, AdipoGen), rabbit anti–myosin-Va (IF 1:200; NBP1-92156, Novus Biologicals), rabbit anti-GAPDH (WB 1:5,000; 60004-1-Ig, Proteintech), mouse IgG2a anti-Arl13b (IF 1:500; 75-287, Antibodies Incorporated), rabbit anti-TOMM20 (IF 1:1,000; ab56783, Abcam), rabbit anti-ARMC9 (WB 1:500 in 5% BSA; HPA026671, MilliporeSigma), mouse IgG2b anti–8-oxo-Dg (IF 1:250; 4354-MC-050, R&D Systems), mouse anti-FLAG (WB 1:5,000; F3165, MilliporeSigma), rabbit anti-NPHP1 (IF 1:1,000; SAB1401267, MilliporeSigma), rabbit anti-Myc (WB 1:1,000; 2278, Cell Signaling Technology), mouse IgG1 anti–α-tubulin (WB 1:1,000; T6199, MilliporeSigma), mouse IgG1 anti-ZN5 (IF 1:1,000; ZDB-ATB-081002-19, Zebrafish International Resource Center, Eugene, Oregon, USA), mouse IgG1 anti-rhodopsin (IF 1:500; ab5417, Abcam), rabbit anti-recoverin (IF 1:500; AB5585-I, MilliporeSigma), rabbit anti-GAPDH (WB 1:1,000; 2118S, Cell Signaling Technology), rabbit anti-IFT88 (IF 1:1,000; 13967-1-AP, Proteintech), rabbit anti-CEP97 (WB 1:1,000, IF 1:1,000; 22050-1-AP, Proteintech), rabbit anti-CEP290 (WB 1:1,000, IF 1:1,000; A301-659A, Thermo Fisher Scientific), rabbit anti-MKS3 (IF 1:250; 13975-1-AP, Proteintech), rabbit anti-TCTN2 (IF 1:250; 17053-1-AP, Proteintech), rabbit anti-MKS1 (IF 1:200; 16206-1-AP, Proteintech), rat anti-ARMC9 (IF 1:200; 90703, BiCell Scientific), mouse IgG1 anti-Zrp1 (IF 1:1,000; ZDB-ATB-081002-43, Zebrafish International Resource Center), MitoTracker Red CMXRos (M7512, Thermo Fisher Scientific).

### Transient transfection.

Transient transfections were performed using TurboFect Transfection Reagent (Thermo Fisher Scientific, R0533). Three million 293T cells were plated on 60 mm culture dishes overnight. Cells were transfected with 2.5 μg of expression constructs according to the manufacturer’s instructions. Cells were harvested 48 hours after transfection.

### Lentivirus production and infection.

Five hundred thousand 293FT cells were plated on 60 mm dishes using TurboFect Transfection Reagent with the following plasmids: 1.5 μg of V-SVG, 2.5 μg pCMV-gag-pol, and 3.5 μg of the pCS-based constructs. The supernatant containing viral particles was harvested 48 hours after transfection. Virus-containing medium was passed through a 0.45 μm filter (Fisher Scientific, 13-100-105). For the infection of RPE1 cells, human conjunctival fibroblasts, and JBTS conjunctiva cells, 5 × 10^5^ cells were seeded onto a 60 mm plate the night before infection and incubated with 3 mL of viral stock. The medium was changed to fresh culture medium 24 hours after infection. From 2 days after infection, cells were maintained in culture medium.

### Generation of NDUFAF2- and ARMC9-knockout RPE1 cells.

The targeting sequences for NDUFAF2 and ARMC9 were at exon 1 of NDUFAF2 (5′-GCAGTACAAGAACTGGAGAG-3′) and exon 3 of ARMC9 (5′-TTTCAAGTTCCATCCGAGAT-3′). The target sequences were cloned into the gRNA cloning vector (pSLQ1654) containing Cas9 via the restriction enzyme BbsI (Thermo Fisher Scientific, FD1014). Knockout cells were all obtained through clonal propagation from a single cell. For genotyping, the following PCR primers were used: 5′-TCCAGGATGGAGGCCGACCT-3′ and 5′-TAGTAGGGACCGCGGACGCA-3′ for NDUFAF2 alleles, and 5′-ATGGGGGACATTCTGGCTCAT-3′ and 5′-GAGGGGTGCACCATAGGGTTG-3′ for ARMC9 alleles. PCR products were cloned and sequenced.

### Immunostaining.

Cells were grown on 0.1 mg/mL of poly-l-lysine–coated coverslips and fixed with methanol at −20°C for 15 minutes. Cells were then washed 3 times with PBS and incubated in blocking buffer that contained 3% BSA (wt/vol) and 0.1% Triton X-100 in PBS for 30 minutes at room temperature (RT). Primary antibodies were all diluted in the blocking buffer and incubated for 2 hours at RT. Alexa Fluor 488–, 594–, or 647–conjugated goat secondary antibodies were used at 1:500 dilution (Thermo Fisher Scientific) and incubated for 1 hour at RT. DNA was visualized using DAPI (Thermo Fisher Scientific). Coverslips were mounted on the slides with mounting medium (ProLong Gold Antifade, Thermo Fisher Scientific). Fluorescent images were obtained using an LSM880 Zeiss confocal microscope. Images were acquired and processed by ZEN software (Carl Zeiss) or ImageJ software (NIH).

### Immunoblotting.

Cells were washed with ice-cold PBS twice and lysed in ice-cold RIPA lysis buffer (MilliporeSigma, 20-188) that contained protease inhibitor cocktail (Thermo Fisher Scientific, PI78430). Cell debris was removed by centrifugation at 13,500*g* for 15 minutes at 4°C. Protein concentrations were determined by the BCA Protein Assay (Thermo Fisher Scientific, 23227). Equal amounts of proteins were mixed with SDS sample buffer, boiled at 95°C for 5 minutes, and separated by SDS-PAGE. The resolved proteins were then transferred onto 0.2 μm nitrocellulose membranes (Bio-Rad, 1620097). Blots were blocked for 1 hour at RT with 5% nonfat milk in TBS-T (20 mM Tris, pH 7.6, 137 mM NaCl, and 0.1% Tween-20) and incubated overnight at 4°C with primary antibodies in blocking solution. Membranes were washed 3 times with TBS-T and incubated with HRP-conjugated anti-mouse or anti-rabbit secondary antibodies for 1 hour at RT (Invitrogen, 31430 and 31460). After washing 3 times with TBS-T, proteins were visualized with ECL Western blotting substrate (Thermo Fisher Scientific, 34095).

### Immunoprecipitation.

Transfected cells were lysed on 60 mm dishes in buffer that contained 50 mM Tris-HCl (pH 8.0), 150 mM NaCl, 1% Nonidet P-40, and 0.1% SDS together with phosphatase and protease inhibitor. One milligram of cell lysates were suspended in 1 mL lysis buffer and incubated with 5 μL of anti-FLAG M2 beads (MilliporeSigma) overnight at 4°C under gentle rotation. Beads were then washed 3 times with lysis buffer that contained protease inhibitors. The immunocomplex was eluted by SDS sample buffer and separated by gels for Western blot analysis.

### Quantification of immunostaining images.

ZEN software (Carl Zeiss) was used to quantify the fluorescent intensity of proteins at the centrioles and transition zone and to quantify cilia length. All cells were treated the same during immunostaining and image acquisition. The same setting was applied to all images. A circle was drawn around the centrioles and transition zone, and the total pixel value of the marked region was measured. The signal ratio of the marked region over the proteins at the centrioles was then normalized to the control group. All quantifications were obtained from at least 3 experiments.

### Injection of morpholino antisense oligonucleotides into zebrafish embryos.

Wild-type zebrafish embryos in the single-cell stage were washed and collected in E3 water (water containing 5 mM NaCl, 0.17 mM KCl, 0.33 mM CaCl_2_, 0.33 mM MgSO_4_) in a Petri dish. Two morpholinos (MOs) were purchased from Gene Tool Company: NDUFAF2-MO1, targeting the translation 5′-UTR of NDUFAF2 (5′-TTGTACTGCACATGCAAACACGTTC-3′), and NDUFAF2-MO2, targeting the translation start codon site of NDUFAF2 (5′-CAATGCGGCTCATCTCTGTGATTTA-3′). 0.4 mM morpholinos and 100 ng/μL RNA with a final concentration of 0.1% phenol red (MilliporeSigma, P0290) were injected into single-cell-stage embryos at the desired concentrations using a PLI-100A microinjector (Harvard Medical Apparatus). After microinjection, the embryos were placed in a Petri dish with E3 medium and incubated for development at 28°C.

### In vitro microinjection of transcribed mRNA into zebrafish embryos.

The cDNA of NDUFAF2 was cloned into a pcDNA3.1 vector, and large amounts of capped RNA were synthesized in vitro using the mMESSAGE mMACHINE T7 Transcription Kit (Invitrogen Ambion, AM1344M). RNA samples were then purified with the RNeasy Mini Kit (Qiagen, 74104). On the day of the injection, 100 ng/μL RNA samples were thawed, vortexed lightly, and spun down briefly with a final concentration of 0.1% phenol red (MilliporeSigma, P0290).

### TEM imaging.

HConF and JBTS cells were grown on Aclar film–based (Electron Microscopy Sciences) coverslips and fixed in 4% paraformaldehyde (Electron Microscopy Sciences, 15710) and 2.5% glutaraldehyde (MilliporeSigma, G5882) in PBS buffer at 37°C for 30 minutes. Cells were then further postfixed in 1% osmium tetroxide (OsO_4_) in PBS buffer for 30 minutes on ice. After dehydrating in a graded series of ethanol, the cells were then infiltrated and embedded in EPON812 resin (Electron Microscopy Sciences, catalog 14120) to generate a resin sample. A microtome (Ultracut UC6, Leica) was used to cut the sample into serial sections (~90 nm thickness), which were then stained with 1% uranyl acetate and 1% lead citrate. Samples were imaged using the JEOL JEM1400 transmission electron microscope with a LaB6 emitter.

### Real-time PCR.

Real-time PCR was carried out in an Applied Biosystems Inc. StepOne Real-Time PCR System using the Maxima SYBR Green/ROX qPCR Master Mix (2×) (Thermo Fisher Scientific, K0221). Primers for 1 rhodopsin marker, 2 transducin markers (*gnat1* and *gnat2*), and 1 nuclear DNA marker (*β*-*actin*) were used. Amplifications were performed in 20 μL reaction mixtures consisting of 100 ng total DNA, 1× SYBR Green PCR Master Mix with 0.5 μM of each primer. Triplicate reactions were performed for each marker in a 96-well plate using a 3-step amplification program of initial denaturation at 95°C for 5 minutes, followed by 40 cycles of 95°C for 15 seconds, 60°C for 30 seconds, and 72°C for 30 seconds. The comparative cycle threshold (Ct) method was used to analyze the data by generating relative values of the amount of rhodopsin and transducin. Relative content was calculated after determination of the difference between Ct of the given rhodopsin marker and 2 transducin markers and that of the calibrator nuclear marker β-actin. Each measurement was repeated in triplicate, and a non-template control was included in each experiment. The sequences of each gene were: *rhodopsin* (zebrafish), forward, 5′-AGTCCTGCCCAGACATCTAG-3′; *rhodopsin*, reverse, 5′-GTACTGTGGGTATTCGTATGGG-3′; *gnat1*, forward, 5′-CGTCAAGTTTGTGTTCGATGC-3′; *gnat1*, reverse, 5′-GAGGAAACGAGCTACAAGGAG-3′; *gnat2*, forward, 5′-CAAACCTGACTACCTTCCCAC-3′; *gnat2*, reverse, 5′-TCTTCCTCTCGGACCTCTG-3′; *β*-*actin*, forward, 5′-TGCTGTTTTCCCCTCCATTG-3′; and *β*-*actin*, reverse, 5′-GTCCCATGCCAACCATCACT-3′.

### Oxygen consumption rate.

Oxygen consumption rate (OCR) was measured using a Seahorse Biosciences XFe96 extracellular flux analyzer. 1.25 × 10^5^ cells per well were seeded in XFe96 cell culture plates. Attachment of the cells was monitored after 24 hours, and cells were incubated overnight at 37°C with 5% CO_2_. Before the assay, cells were changed to Seahorse XF DMEM medium with 1 mM pyruvate, 2 mM glutamine, and 10 mM glucose and equilibrated for 1 hour at 37°C without CO_2_. The OCR was measured afterward using the following inhibitors: 2.5 μM oligomycin, 2 μM carbonyl cyanide 4-(trifluoromethoxy)phenylhydrazone (FCCP), and 0.5 μM rotenone with 0.5 μM antimycin A (Agilent Technologies, 103015-100). For each condition, cycles were performed in triplicate with 3 minutes of mixing followed by 3 minutes of measurement. After completion of the assay, the cell number per well was determined using the Cytation 5 (Agilent Technologies). The OCR was normalized to the cell number for each well.

### Catalytic activity of mitochondrial complex I.

The procedure followed the previously published protocol (74). The fibroblast lysate was suspended in 1 mL of 10 mM ice-cold hypotonic Tris buffer (pH 7.6). The suspension was then taken up and expelled several times using a 26 gauge needle until it became a homogeneous solution. After the addition of 200 μL of 1.5 M sucrose, the homogeneous solution was centrifuged at 600*g* for 10 minutes at 4°C. To collect the supernatant, the solution was centrifuged at 14,000*g* for 10 minutes at 4°C, and the mitochondrial pellet was then suspended in 0.5 mL of 10 mM ice-cold hypotonic Tris buffer (pH 7.6). The homogeneous solution was frozen and thawed in liquid nitrogen and at 37°C three times. Enriched mitochondrial fractions were purified from fibroblast lysates. Complex I activity in cells was analyzed by determining rotenone-sensitive activities followed by detection of 340 nm wavelength.

### Assay of NAD^+^/NADH ratio.

The procedure followed the protocol of the Abcam NAD/NADH Assay Kit (ab65348). Two million cells were washed in 1× PBS and lysed in 500 μL buffer (0.25 M sucrose, 10 mM Tris-HCl, pH 7.5, 10 mM KCl, 1.5 mM MgCl_2_, 1 mM EDTA, 1 mM dithiothreitol, and protease inhibitor). Cells were placed on ice for 15 minutes and sheared with a 26 gauge needle, and homogenates were centrifuged at 700*g* for 5 minutes at 4°C. Supernatants were then collected and centrifuged at 10,000*g* for 30 minutes at 4°C. The pellet was the mitochondrial fraction. The pellet was resuspended in 400 μL NADH/NAD extraction buffer, and cells were extracted by 2 freeze/thaw cycles (20 minutes on dry ice followed by 10 minutes at RT). Extracted cells were vortexed for 10 seconds and then centrifuged for 5 minutes at 4°C at top speed in a cold microcentrifuge to remove any insoluble material. The supernatant was separated, and enzyme was removed by passage through a 10kD Spin Column (Abcam, ab93349) before performance of the assay. For the NADH assay, half of the extraction sample was aliquoted and heated at 60°C for 30 minutes. The standard curve was prepared, and samples loaded into 96-well plates according to the manufacturer’s instructions (Abcam, ab65348).

### Image analysis of mitochondrial length.

Mitochondrial dynamics were analyzed from fluorescence microscope images using Mytoe, software developed by Andre Ribeiro’s laboratory (75).

### Proximity ligation assay.

Five hundred thousand cells were seeded on cover slides and fixed with 4% paraformaldehyde in PBS for 20 minutes at RT. For ARMC9 antibody, the initial fixation was followed by fixation in ice-cold 100% methanol for 15 minutes at –20°C. The slides were then washed twice with PBS, and cells were blocked with Duolink block solution (MilliporeSigma, DUO92101-1KT) for 1 hour at RT. During proximity ligation assays (PLAs) (MilliporeSigma, DUO92101), the primary antibodies were diluted in Duolink antibody dilution buffer (MilliporeSigma, DUO92101-1KT) and stained overnight at 4°C. The next day, the slides were washed with a large volume of 5% BSA in PBS for 10 minutes. Before the PLA assay, a 20 μL total volume per reaction of secondary antibodies was prepared with 4 μL Anti–rabbit PLUS, Affinity-purified Donkey Anti–rabbit IgG (H+L) antibody and 4 μL Anti–mouse MINUS, Affinity-purified Donkey Anti–mouse IgG (H+L) antibody plus 12 μL Duolink antibody dilution buffer for 20 minutes at RT. Secondary antibodies were then added onto slides and incubated at 37°C for 1 hour. Before the ligation process of the PLA assay, the slides were washed with 1× of buffer A twice for 5 minutes. During the ligation process, the prepared 20 μL total volume per reaction of ligation mix was added onto slides with 4 μL ligation stock, 15.5 μL double-distilled H_2_O (ddH_2_O), and 0.5 μL ligase for 30 minutes at 37°C. Before the amplification process of the PLA assay, the slides were washed by 1× of buffer A twice for 5 minutes. During the amplification process, the 20 μL total volume per reaction of amplification mix was prepared and added onto slides with 4 μL amplification stock, 15.75 μL ddH2O, and 0.25 μL polymerase for 100 minutes at 37°C. After that, the slides were washed twice with 1× of buffer B for 10 minutes and once with 0.01× of buffer B for 1 minute. After aspiration of buffer B, cells were mounted in ProLong Gold Antifade Reagent with DAPI (MilliporeSigma, DUO82040).

### Fluo-4 AM.

The Fluo-4 AM, cell permeant (F14201, Invitrogen), stock solution was prepared in DMSO at a concentration of 1 mM before loading of the cells with the product. A final concentration of 2 μM in E3 buffer was achieved followed by incubation at 28°C for 15 minutes. Ca^2+^ binding was used to indicate fluorescent signal (excitation/emission peaks at 494/506 nm), with detection in the time-lapse live images under a confocal microscope.

### Study subjects, skin biopsy, and fibroblast culture.

Family 1 was identified at Indiana University, and family 2 was identified at Lucile Packard Children’s Hospital Stanford. A fibroblast sample was harvested from surgical waste (removed conjunctiva) from the proband in family 2. The biopsy was minced, transferred to the bottom of a T-25 culture flask, and cultured in minimum essential medium supplemented with 10% FBS, 1% non-essential amino acids, and 1% antibiotics/antimycotics. Cultures were maintained at 37°C in a 5% CO_2_ and 5% O_2_ incubator, and medium was replaced after 1 week. Once the fibroblasts had grown to approximately 50% confluence, cells were trypsinized and replated into a T-75 flask. Cells were then collected and frozen at –80°C.

### Functional measurements of ocular motility.

Eye movements were quantified using Tobii Pro Spectrum (Tobii Technology AB) infrared video oculography. This system uses dual near-infrared cameras to sample the position of the subject’s eyes at a rate of 1,200 Hz with an accuracy of 0.3°. The subject was positioned 61 cm from the screen, without a chin rest. A child-appropriate visual stimulus pattern was used with the Tobii Pro Lab software on a 24-inch monitor (resolution 1,920 × 1,080 pixels). A stimulus target of a small cartoon animal spanning 128 by 165 pixels was placed in the center of the monitor to induce fixation and then moved to each of 4 eccentric positions (252 pixels in 4 cardinal positions from center fixation) to stimulate reflexive saccades. After the eye tracking recording, fixation and saccade data were analyzed using Tobii Pro Lab software and oculometrics quantified in 5 areas of interest (AOIs; Tobii Pro Lab), including gaze duration, fixation count, saccade count, and scan pattern.

### Statistics.

All data are presented as mean with standard deviation (SD) from at least 3 independent experiments (GraphPad Prism 8). Experimental samples and numbers for statistical testing are reported in the corresponding figure legends. To evaluate the differences among 3 or more groups, 1-way ANOVA was used, followed by a Tukey-Kramer multiple-comparison test. For 2-group comparisons, a 1-tailed Student’s *t* test was used for normally distributed data. *P* values less than 0.05 were considered to indicate statistical significance.

### Study approval.

The families consented to studies approved by the Indiana University Institutional Review Board (IRB 3023) and Stanford University Institutional Review Board (IRB-32223) and gave written permission to publish photographs (where applicable) and the pertinent clinical information. All study participants were informed of the purpose of the examination, and all the participating individuals signed a written informed consent prior to the study, ensuring their voluntary participation. The overall study was approved by the Institutional Review Board of Stanford University for studies involving humans (IRB-5537 and IRB-39756) and adhered to the tenets of the Declaration of Helsinki and ethical guidelines and protocols to protect the participants’ privacy and well-being.

### Data availability.

All the data sets generated for this study can be found within the article figures and supplemental material. Values for all data points in graphs are reported in the Supporting Data Values file.

## Author contributions

CHL designed and carried out all experiments and data analysis and wrote the manuscript. ZL, SC, YH, YS, and WJW contributed to manuscript editing. FL contributed to setting up injections in zebrafish. TJK and KN contributed to setting up cryosections in zebrafish. ARB, CQY, and EBK provided patient samples. YJL provided valuable advice and experimental suggestions. YS supervised the project.

## Supplementary Material

Supplemental data

Unedited blot and gel images

Supplemental video 1

Supplemental video 2

Supplemental video 3

Supplemental video 4

Supporting data values

## Version 1 07/01/2024

Electronic publication
