# Supplementary material for: Primary cilia formation requires the Leigh syndrome–associated mitochondrial protein NDUFAF2
Source: J Clin Invest. 2024 Jul 1;134(13):e175560. doi: 10.1172/JCI175560 (PMC11213510; doi:10.1172/JCI175560)
Supplement: Supplemental data [file jci-134-175560-s176.pdf]

Supplemental Figure

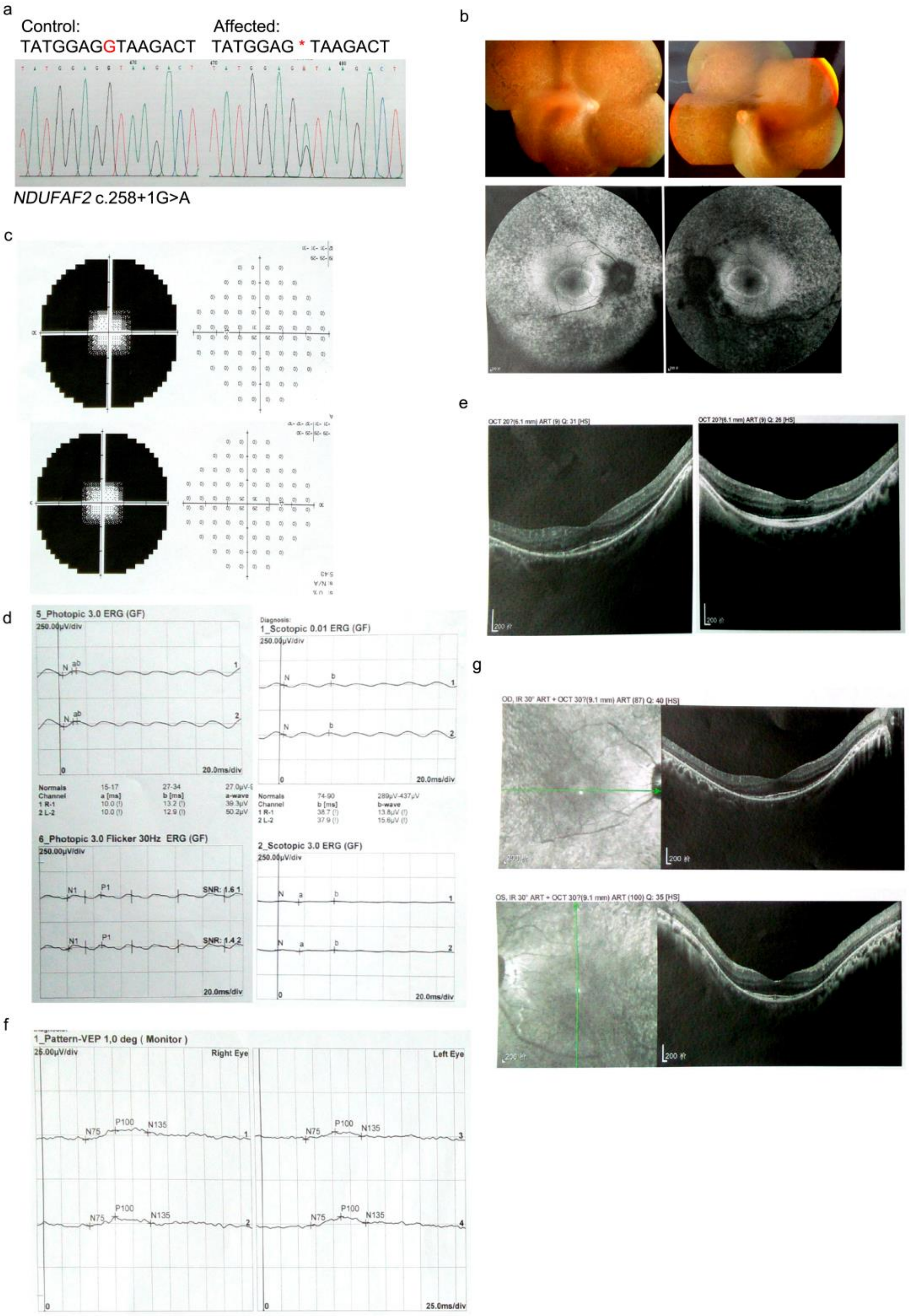

Supplemental Figure 1. NDUFAF2 mutation in a patient with bilateral retinal degeneration and optic nerve atrophy. (a) A 28-yo woman presented with a novel NDUFAF2 mutation c.258+1 G>A (b) Color fundus photography of both eyes showing pale optic nerve, attenuated blood vessels, and pigmentary retinopathy. (c) Red-free fundus imaging showing bilateral diffuse pigmentary degeneration with residual bullseye lesion in central macula. (d) Humphrey visual field 24-2 demonstrating severe

constriction with preservation of central island in both eyes. (e) Phototopic and scotopic electroretinogram showing flat amplitude and decreased a- b- waves in both eyes. (f) Ocular coherence tomography showing loss of photoreceptors and thinning of retinal ganglion cell layer in both eyes. (g) Visual evoked potential (VEP) showing decreased P100 amplitude in both eyes.

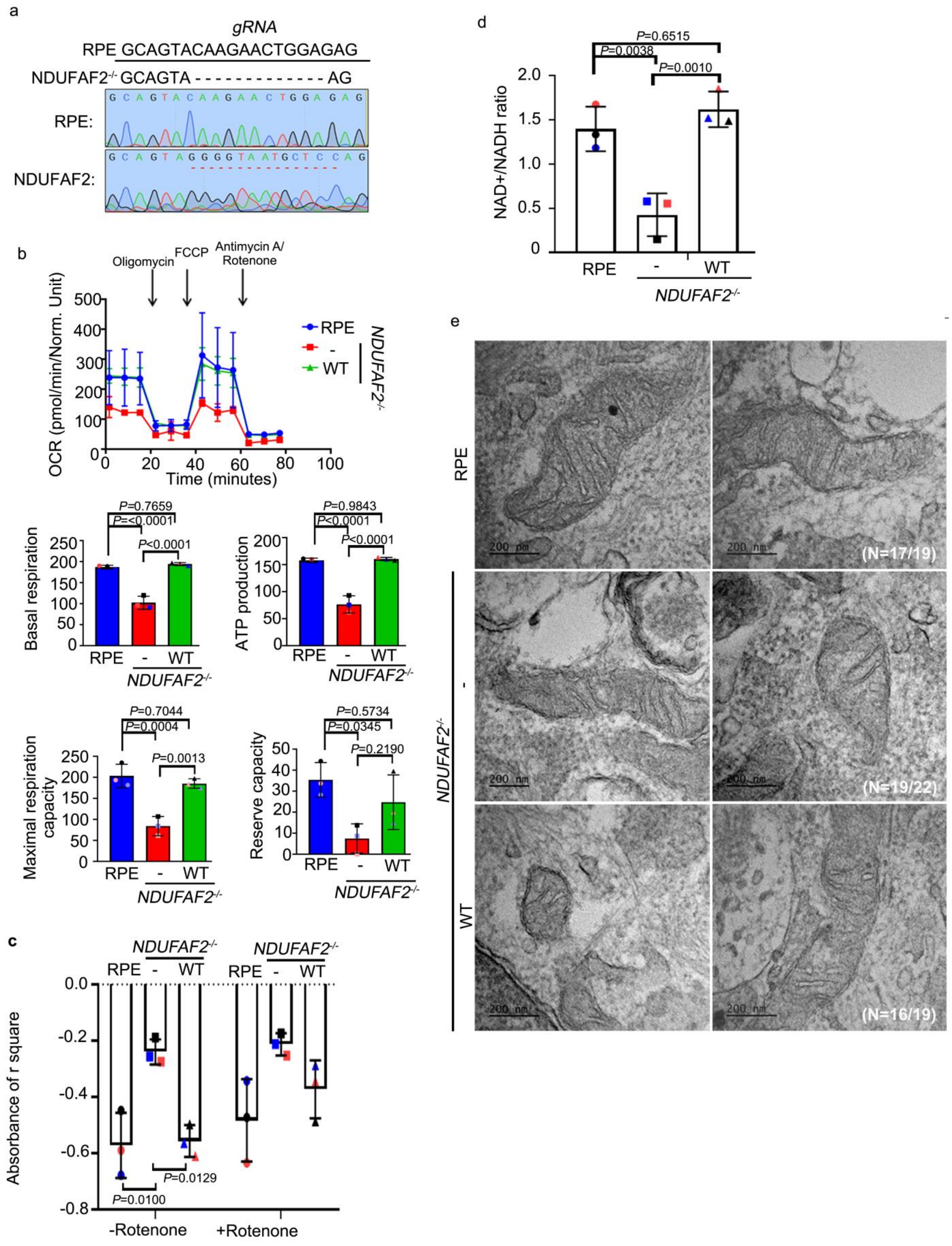

Supplemental Figure 2. Loss of NDUFAF2 affects mitochondrial activity. (a) Editing sequencing outcomes in $NDUFAF2^{-/-}$ cells. The upstream is the sequence of RPE1 cells; the downstream is the sequence of two different *NDUFAF2*$^{-/-}$ cells. (b) Mitochondrial activity in wild-type, *NDUFAF2*$^{-/-}$, and $NDUFAF2^{WT}$ -re-expressing RPE1 cells. The oxygen consumption rate was measured by Seahorse Analyzer. (c) Catalytic enzyme activity of complex I in purified mitochondria of wild-type, *NDUFAF2*$^{-/-}$, and $NDUFAF2^{WT}$ -re-expressing RPE1 cells. (d) NAD+/NADH ratio in

wild-type, *NDUFAF2*$^{-/-}$, and NDUFAF2^{WT} -re-expressing RPE1 cells. (e) TEM images of wild-type, NDUFAF2$^{-/-}$, and NDUFAF2^{WT} -re-expressing RPE1 cells showing mitochondrial cristae. Bars in each graph represent mean ± SD. Exact *P* values are indicated. ANOVA followed by a Tukey-Kramer multiple-comparison test.

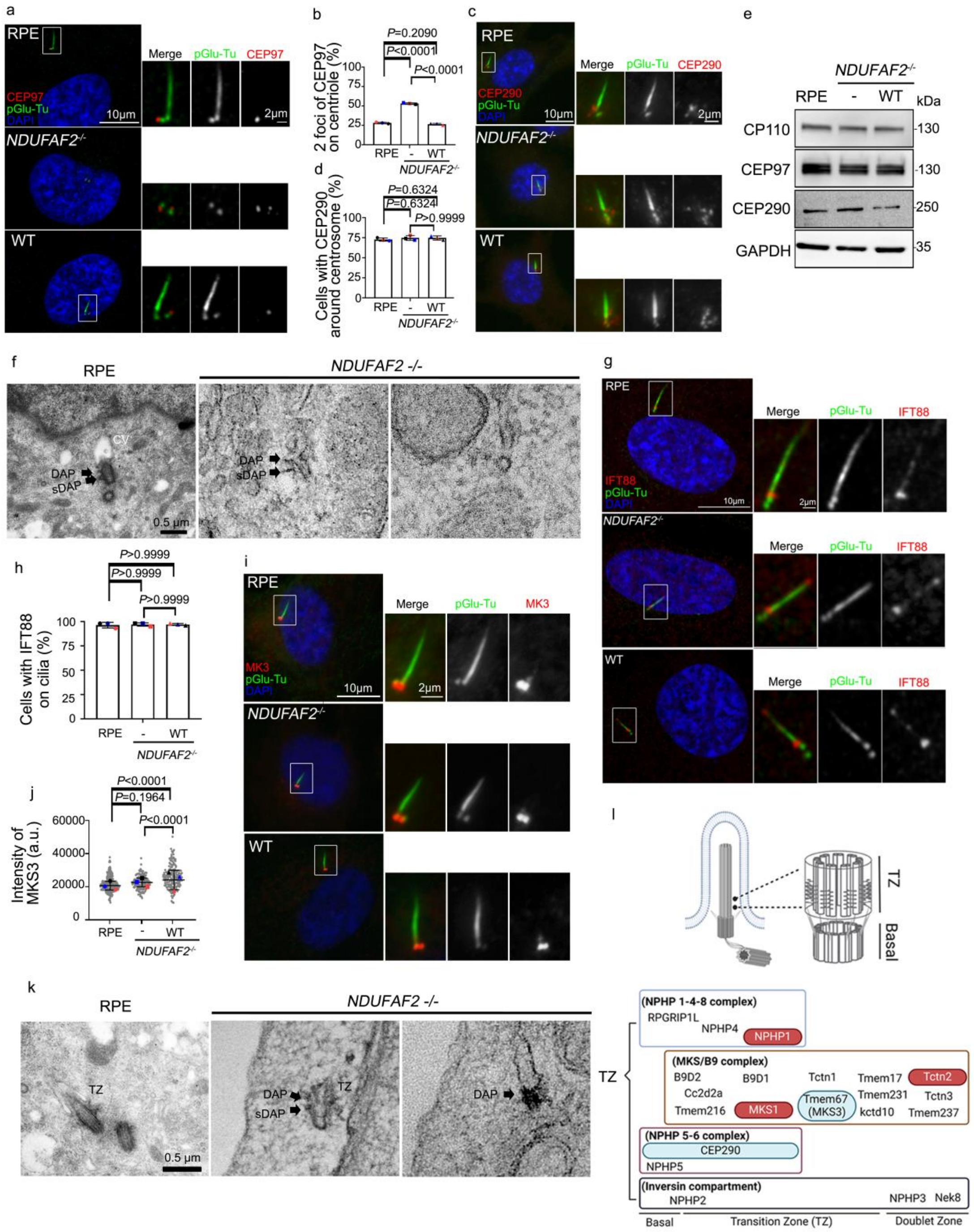

Supplemental Figure 3. Loss of NDUFAF2 in RPE cells results in transition zone defects. (a) Immunofluorescent analysis of cells serum starved for 2 days. Cells stained with CEP97 (red) and pGlu-Tu (green) antibodies. DNA (DAPI, blue). Scale bars as indicated. (b) Graph shows the percentage of serum-starved cells with two CEP97 dots at the centrioles. > 150 cells analyzed for each independent experiment. (c) Immunofluorescent analysis of cells serum starved for 2 days. Cells stained with CEP290 (red) and pGlu-Tu (green) antibodies. DNA (DAPI, blue). Scale bars as

indicated. (d) Graph shows the percentage of serum-starved cells with CEP290 signals around the centrioles. > 150 cells analyzed for each independent experiment. (e) Western blot analysis was performed with antibodies against CP110, CEP97, CEP290 and GAPDH. (f) TEM images of wild-type and NDUFAF2$^{-/-}$ RPE1 cells showing centriole and ciliary vesicle. (g) Immunofluorescent analysis of cells serum starved for 2 days. Cells stained with IFT88 (red) and pGlu-Tu (green) antibodies. DNA (DAPI, blue). Scale bars as indicated. (h) Graph shows the percentage of serum-starved cells with IFT88 signals on the cilium. > 150 cells analyzed for each independent experiment. (i) Immunofluorescent analysis of cells serum starved for 2 days. Cells stained with MKS3 (red) and pGlu-Tu (green) antibodies. DNA (DAPI, blue). Scale bars as indicated. (j) Quantification of MKS3 signal intensity at the centrioles. > 100 cells analyzed for each independent experiment. (k) TEM images of wild-type and NDUFAF2$^{-/-}$ RPE1 cells showing centriole and transition zone (TZ). (l) Schematic diagram showing the localization of transition zone. Red means affected protein in *NDUFAF2*$^{-/-}$ RPE cells and blue means unaffected protein in *NDUFAF2*$^{-/-}$ RPE cells. It is generated by BioRender. Bars in each graph represent mean ± SD. Exact *P* values are indicated. ANOVA followed by a Tukey-Kramer multiple-comparison test.

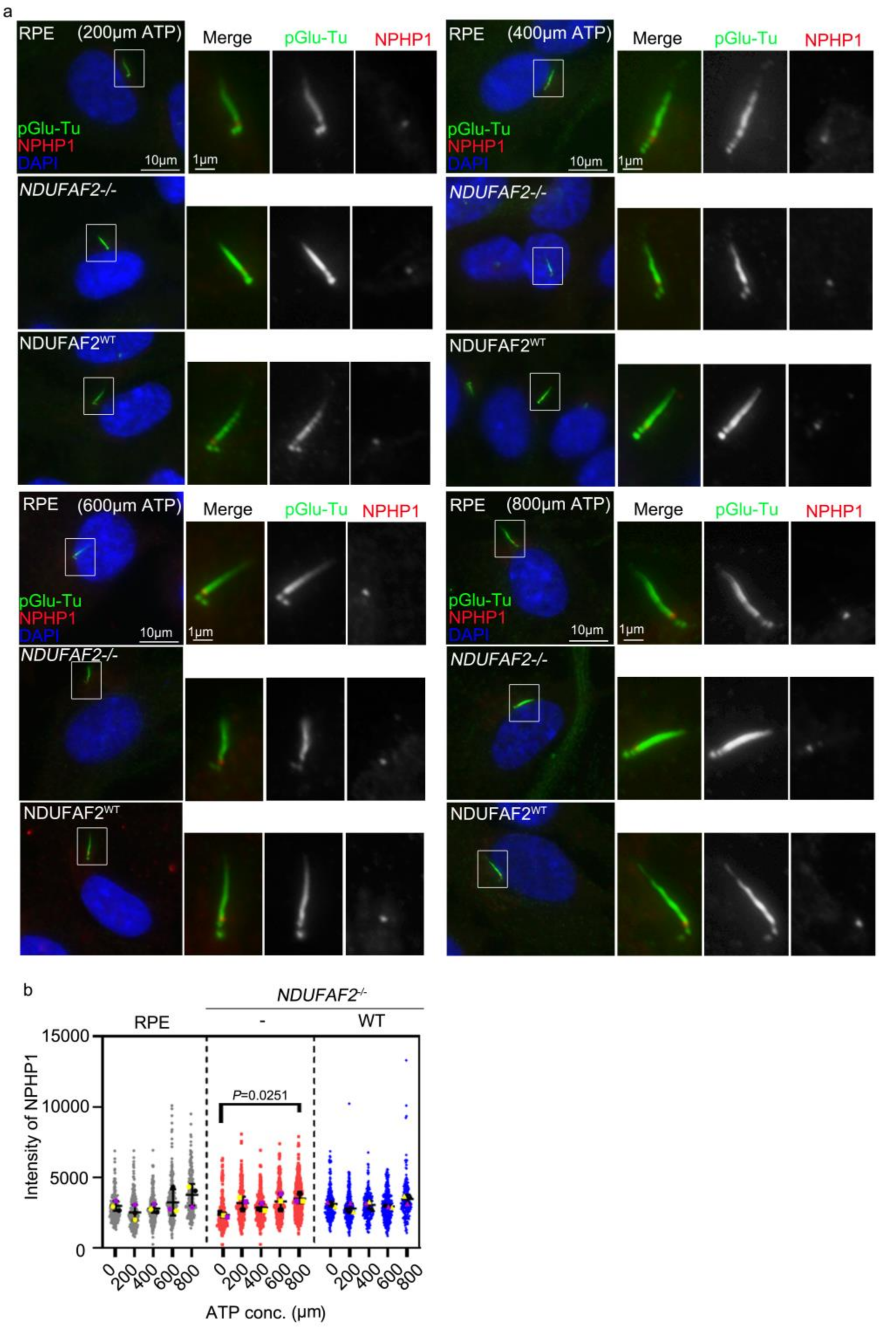

Supplemental Figure 4. ATP promotes transition zone formation (a) Immunofluorescent analysis of cells serum starved for 2 days. Cells stained with NPHP1 (red) and pGlu-Tu (green) antibodies. DNA (DAPI, blue). Scale bars as indicated. (b) Quantification of the intensity of NPHP1 signals at the centrioles. > 150 cells analyzed for each independent experiment. Bars in each graph represent mean ± SD. Exact *P* values are indicated. ANOVA followed by a Tukey-Kramer multiple-comparison test.

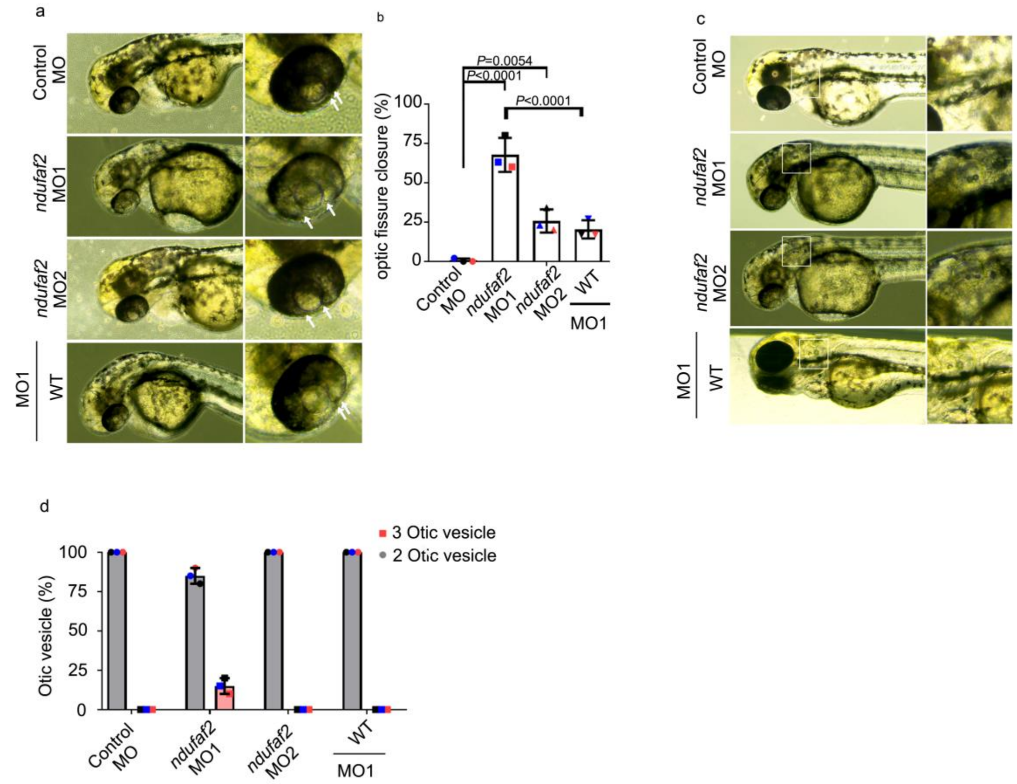

Supplemental Figure 5. NDUFAF2 affects optic fissure closure but does not affect the number of otic vesicles. (a) Transmitted light images of eyes in control MO, *ndufaf2* morpholino-injected zebrafish larvae and *NDUFAF2*^{WT} re-expressing in *ndufaf2* morpholino-injected zebrafish larvae at 3 dpf. Arrows indicate the margins of the optic fissure. (b) Quantification of optic fissure closure in control MO, ndufaf2 morpholino-injected zebrafish larvae and *NDUFAF2*^{WT} re-expressing in ndufaf2 morpholino-injected zebrafish larvae at 3 dpf. > 30 larvae analyzed for each independent experiment. (c) Transmitted light images of eyes in control MO, *ndufaf2* morpholino-injected zebrafish larvae, and *ndufaf2* morpholino-injected zebrafish larvae and *NDUFAF2*^{WT} re-expressing at 2 dpf. Arrows point to otic vesicle margins. (d) Quantification of optic fissure closure in control MO, *ndufaf2* morpholino-injected zebrafish larvae, and *ndufaf2* morpholino-injected zebrafish larvae and *NDUFAF2*^{WT} re-expressing at 2 dpf. > 30 larvae were analyzed for each independent experiment. Bars in each graph represent mean ± SD. Exact *P* values are indicated. ANOVA followed by a Tukey-Kramer multiple-comparison test.

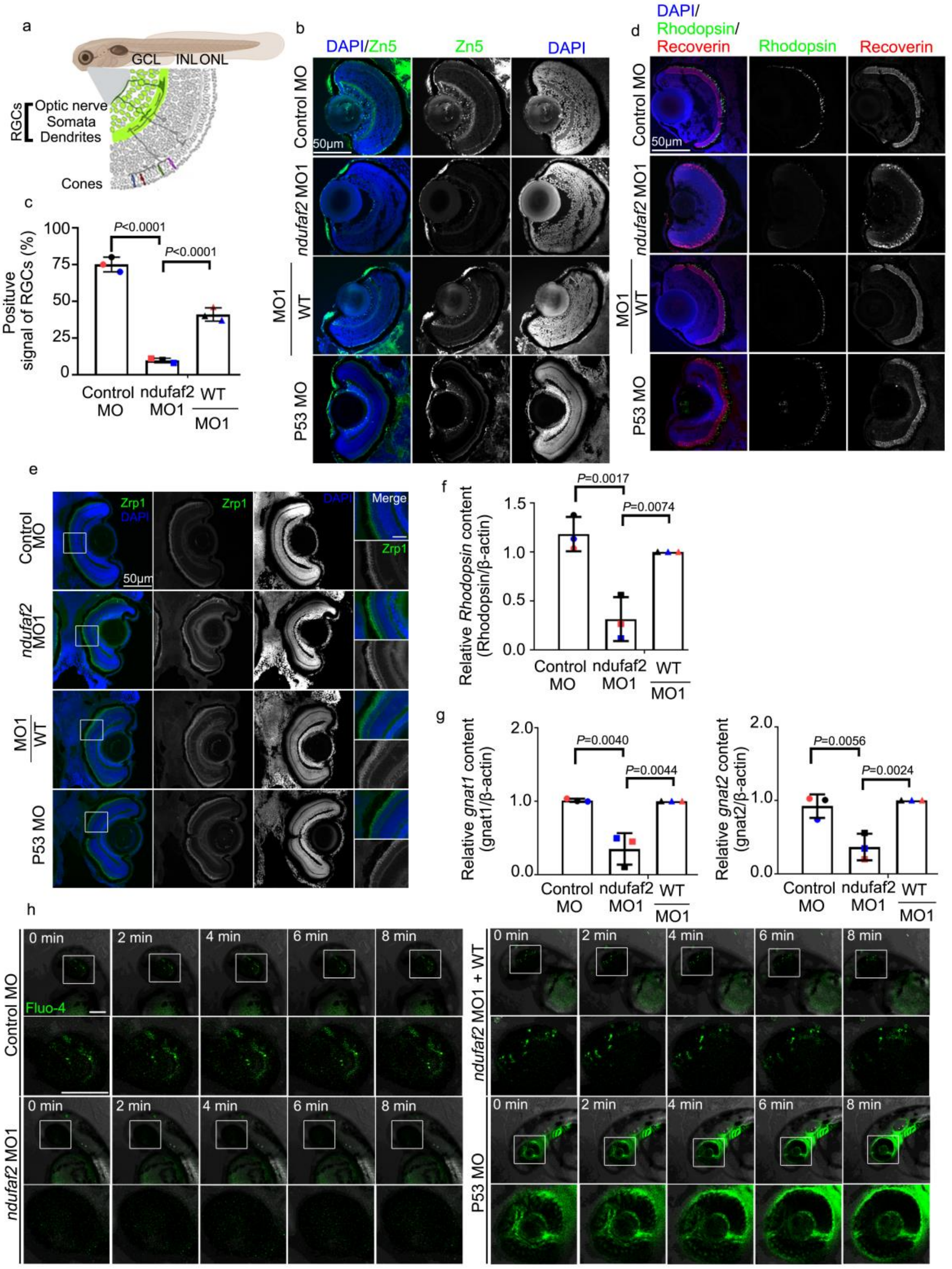

Supplemental Figure 6. Photoreceptor dysfunction and degeneration in zebrafish *ndufaf2* morpholino-injected mutants. (a) Schematic diagram showing photoreceptor structure in zebrafish. It is generated by BioRender. (b) Light microscopic images showing retinal ganglion cells (RGC) in zebrafish larvae at 5 dpf. Zebrafish larvae are stained with Zn5 (green), DNA is stained with DAPI (blue). Scale bars as indicated. (c) Quantification of RGC signals in the GCL region of zebrafish larvae at 5 dpf. 10 larvae analyzed for each independent experiment. (d)

Light microscopic images showing photoreceptors in zebrafish larvae at 5 dpf. Zebrafish larvae are stained with rhodopsin (green) and recoverin (red), DNA is stained with DAPI (blue). Scale bars as indicated. (e) Light microscopic images showing cone photoreceptor in zebrafish larvae at 5 dpf. Zebrafish larvae are stained with Zrp1 (green), DNA is stained with DAPI (blue). Scale bars as indicated. (f) Quantitative real-time PCR (RT-PCR) validation of rhodopsin in zebrafish larvae at 5 dpf. RT-PCR was repeated three times with different batches. Gene expression values are normalized to β-actin. (g) Quantitative real-time PCR (RT-PCR) validation of transducin (*gnat1 and gnat2*) in zebrafish larvae at 5 dpf. RT-PCR was repeated three times with different batches. Gene expression values are normalized to β-actin. (h) Zebrafish larvae at 5 dpf were incubated at 28℃ for 45 min with Fluo-4, calcium indicator. Bars in the graph represent mean ± SD. Exact *P* values are indicated. ANOVA followed by a Tukey-Kramer multiple-comparison test.

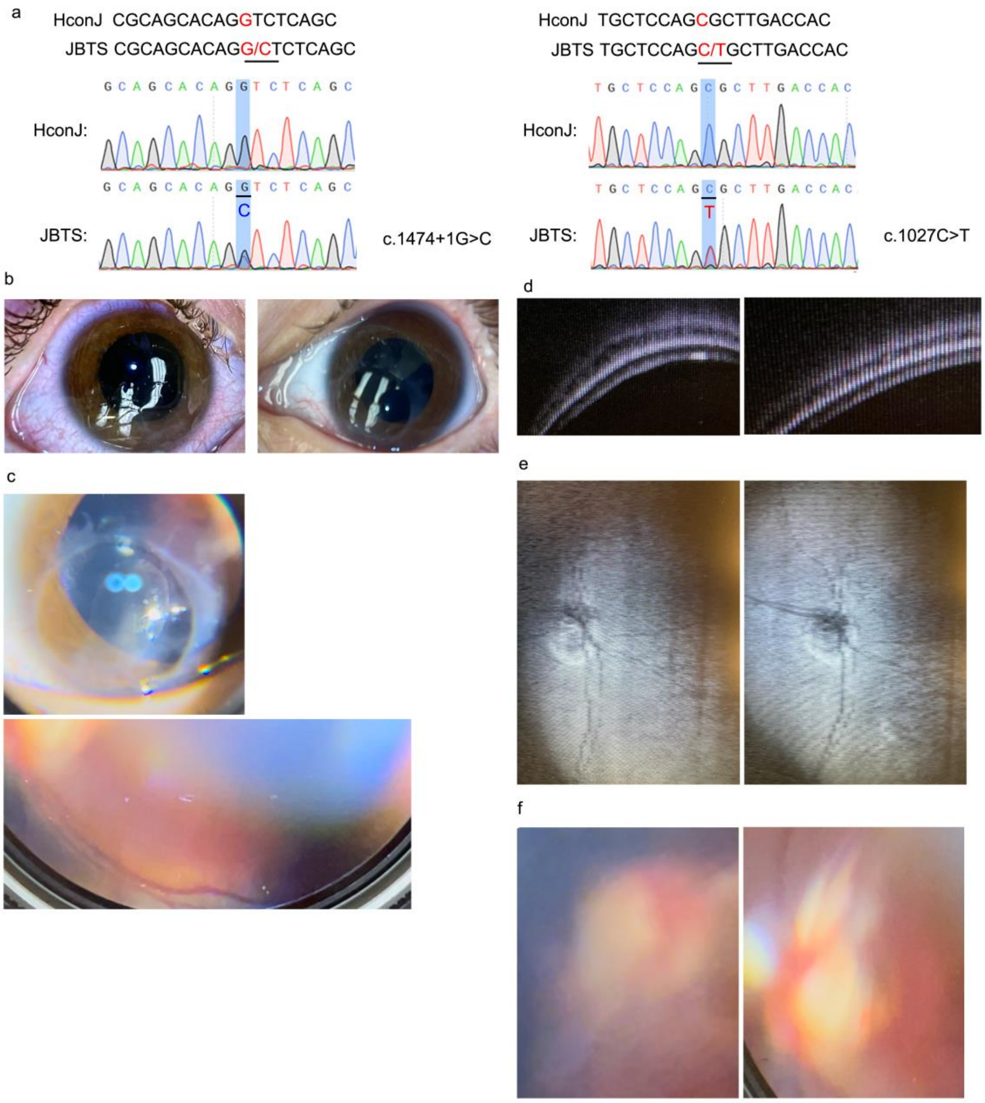

Supplemental Figure 7. ARMC9 mutation-associated midbrain and retinal defects. (a) The child carries ARMC9 mutations (case number UW349-3, c.1474+1G>C and c. 1027 C>T) (b) Anterior segment photos showing corneal exposure keratitis in right eye and inferior corneal transplant in left eye. (c) Retinal photograph of left eye showing early pigmentary retinal changes. (d) and (e) Ocular coherence tomography of right eye showing attenuated blood vessels. (f) Optic nerve

head photographs showing cupping of optic nerve in both eyes and myelinated optic nerve in left eye.

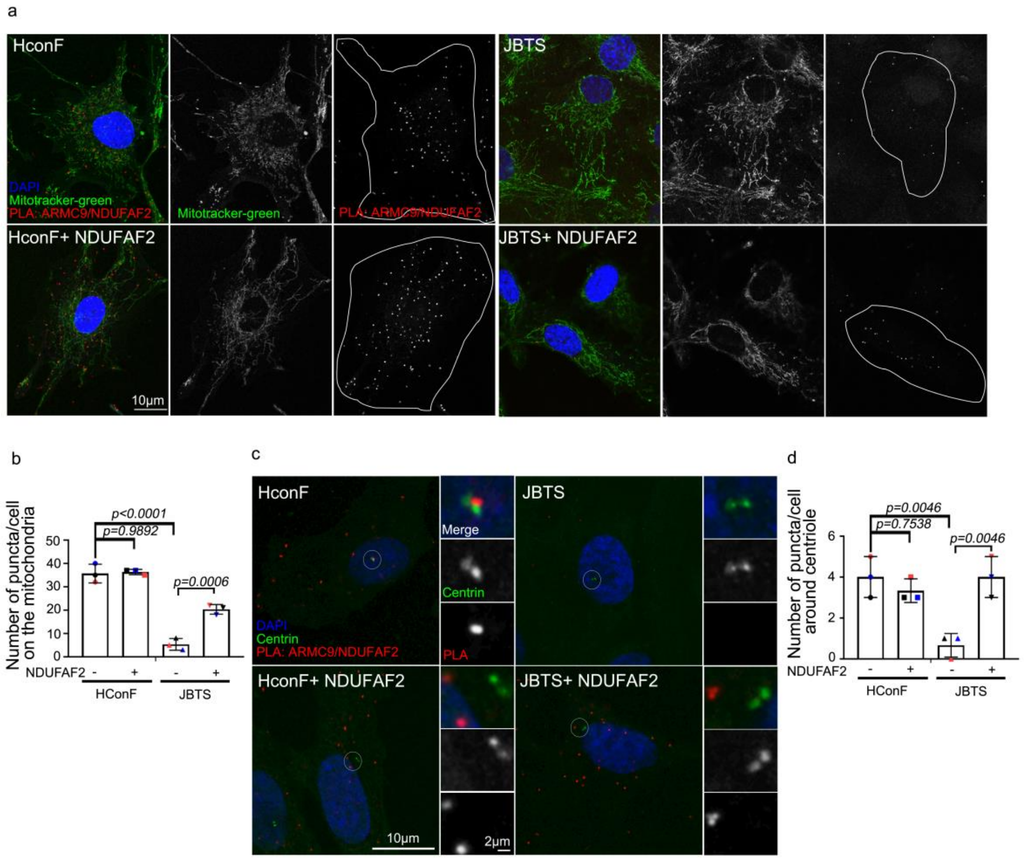

Supplemental Figure 8. The interaction between ARMC9 and NDAUFAF2 is decreased in JBTS patient-derived cells. (a) Cells were incubated with mitochondrial marker in green and with anti-ARMC9 and anti-NDUFAF2 antibodies then examined using PLA probes in red. (b) Quantification of puncta on mitochondria in HConF cells, JBTS cells, and both cell lines overexpressing NDUFAF2.. (c) Cells were incubated with centriole marker in green and with anti-ARMC9 and anti-NDUFAF2 antibodies, then examined using PLA probes in red. (d) Quantification of puncta around centrioles in HConF cells, JBTS cells, and both cell lines overexpressing NDUFAF2. The bars in each graph represent mean ± SD. Exact P values are indicated

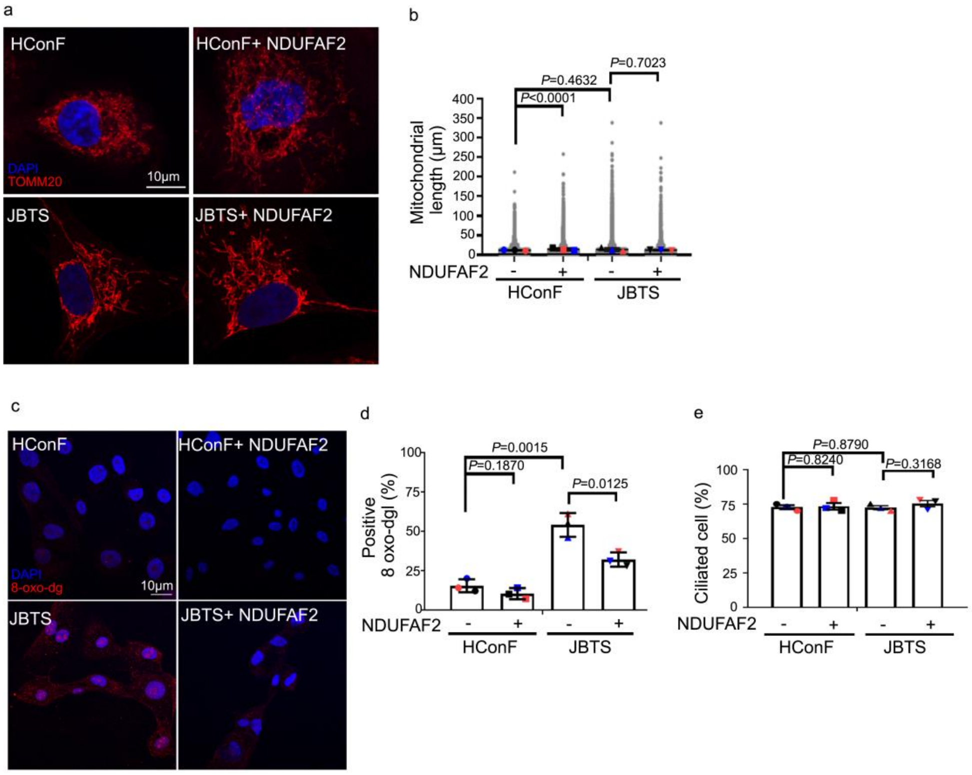

Supplemental Figure 9. NDUFAF2 rescues defective ciliogenesis in JBTS patient-derived cells. (a) Representative cells stained by immunofluorescence for Mitotracker-red (red). Nuclei visualized with DAPI (blue). Scale bars 10 µm. (b) Mitochondrial length measured by Mytoe of HConF cells, JBTS cells, and both cell lines overexpressing NDUFAF2. > 100 cells measured. Bar line represents mean with standard deviation (mean ± SD, Student's t test). (c) Representative cells stained by immunofluorescence for 8-oxo-dg (red). Nuclei visualized with DAPI (blue). Scale bars 10 µm. (d) Quantitation of 8-oxo-dg signal in individual cells. (e) Immunostaining for polyglutamylated tubulin of cells serum starved for 2 days. The percentage of ciliated cells is quantified. > 150 cells analyzed for each independent experiment. Exact *P* values are indicated. ANOVA followed by a Tukey-Kramer multiple-comparison test.

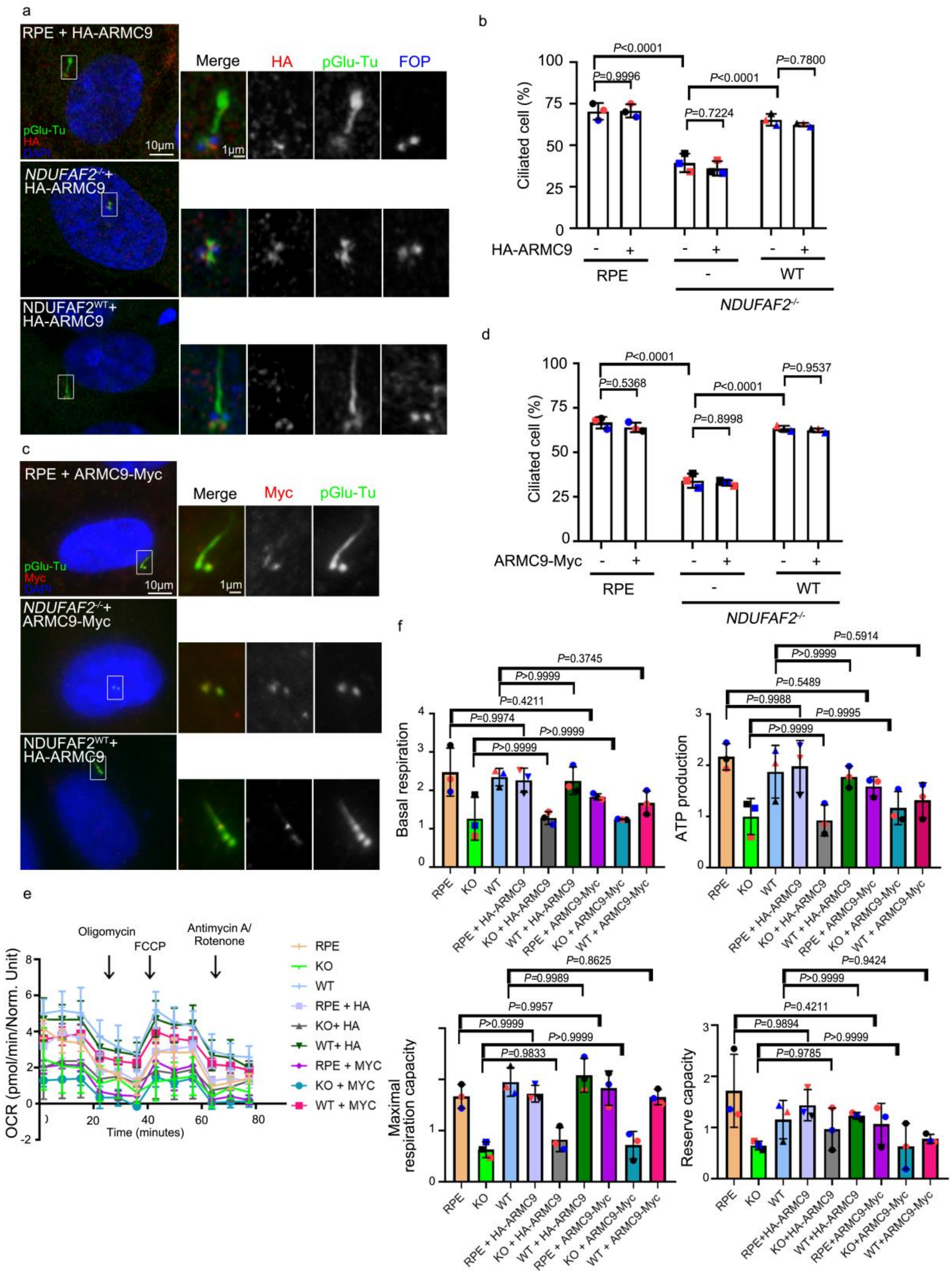

Supplemental Figure 10. Overexpression of ARMC9 is not sufficient for cilia formation. (a) Overexpression of HA tagged of N-terminal of ARMC9 into cells and immunofluorescent analysis of cells serum starved for 2 days. Cells stained with HA (red), pGlu-Tu (green) and FOP (blue) antibodies. DNA (DAPI, blue). Scale bars as indicated. (b) Percentage of ciliated cells after serum starving for 2 days. > 150 cells analyzed for each independent experiment. (c) Overexpression of Myc tagged of C-terminal of ARMC9 into cells and immunofluorescent analysis of cells serum

starved for 2 days. Cells stained with HA (red) and pGlu-Tu (green) antibodies. DNA (DAPI, blue). Scale bars as indicated. (d) Percentage of ciliated cells after serum starving for 2 days. > 150 cells analyzed for each independent experiment. (e) and (f) Oxygen consumption rate of cells measured by Seahorse Analyzer. Exact *P* values are indicated. ANOVA followed by a Tukey-Kramer multiple-comparison test.

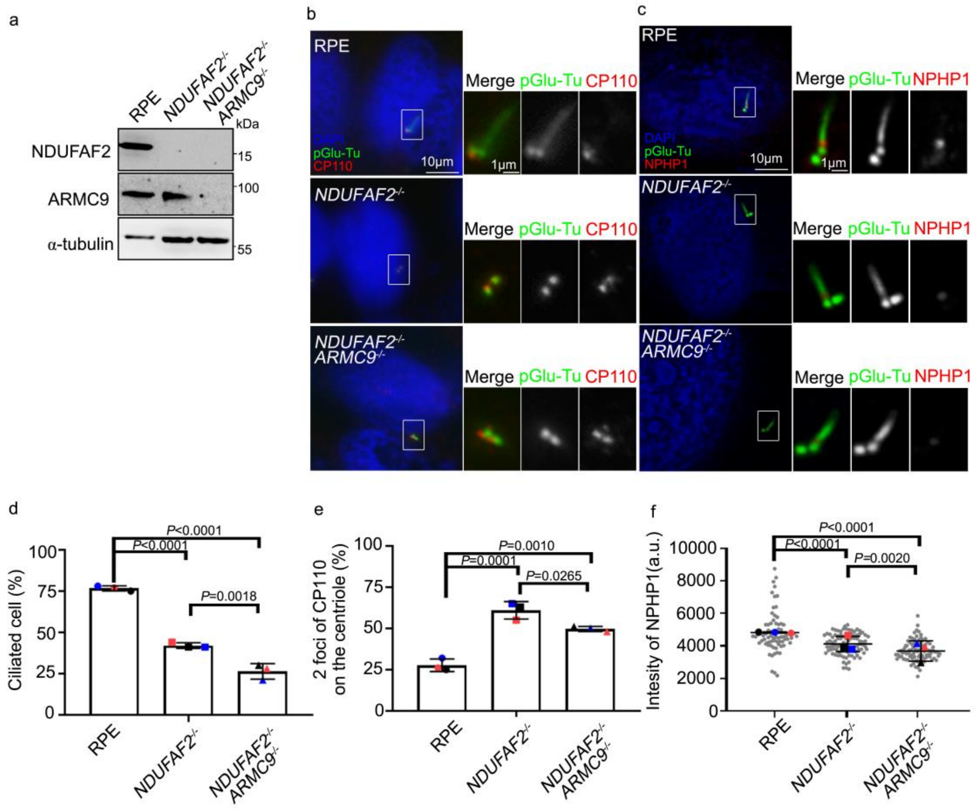

Supplemental Figure 11. ARMC9 is required for cilia formation (a) Double knockout ARMC9-/- NDUFAF2-/- cells. Western blot analysis performed with antibodies against NDUFAF2, ARMC9, and α-tubulin. (b) Immunofluorescent analysis of cells serum starved for 2 days. Cells stained with CP110 (red) and pGlu-Tu (green) antibodies. DNA (DAPI, blue). Scale bars as indicated. (c) Immunofluorescent analysis of cells serum starved for 2 days. Cells stained with NPHP1 (red) and pGlu-Tu (green) antibodies. DNA (DAPI, blue). Scale bars as indicated. (d) Percentage of ciliated cells after serum starving for 2 days. > 150 cells analyzed for each independent experiment. (e) Graph shows the percentage of serum starved cells with two CP110 dots at the centrioles. > 150 cells analyzed for each independent experiment. (f) Quantification of the intensity of NPHP1 signals at the centrioles. > 50 cells analyzed for each independent experiment. The bars in each graph represent mean ± SD. Exact P values are indicated. ANOVA followed by a Tukey-Kramer multiple-comparison test.

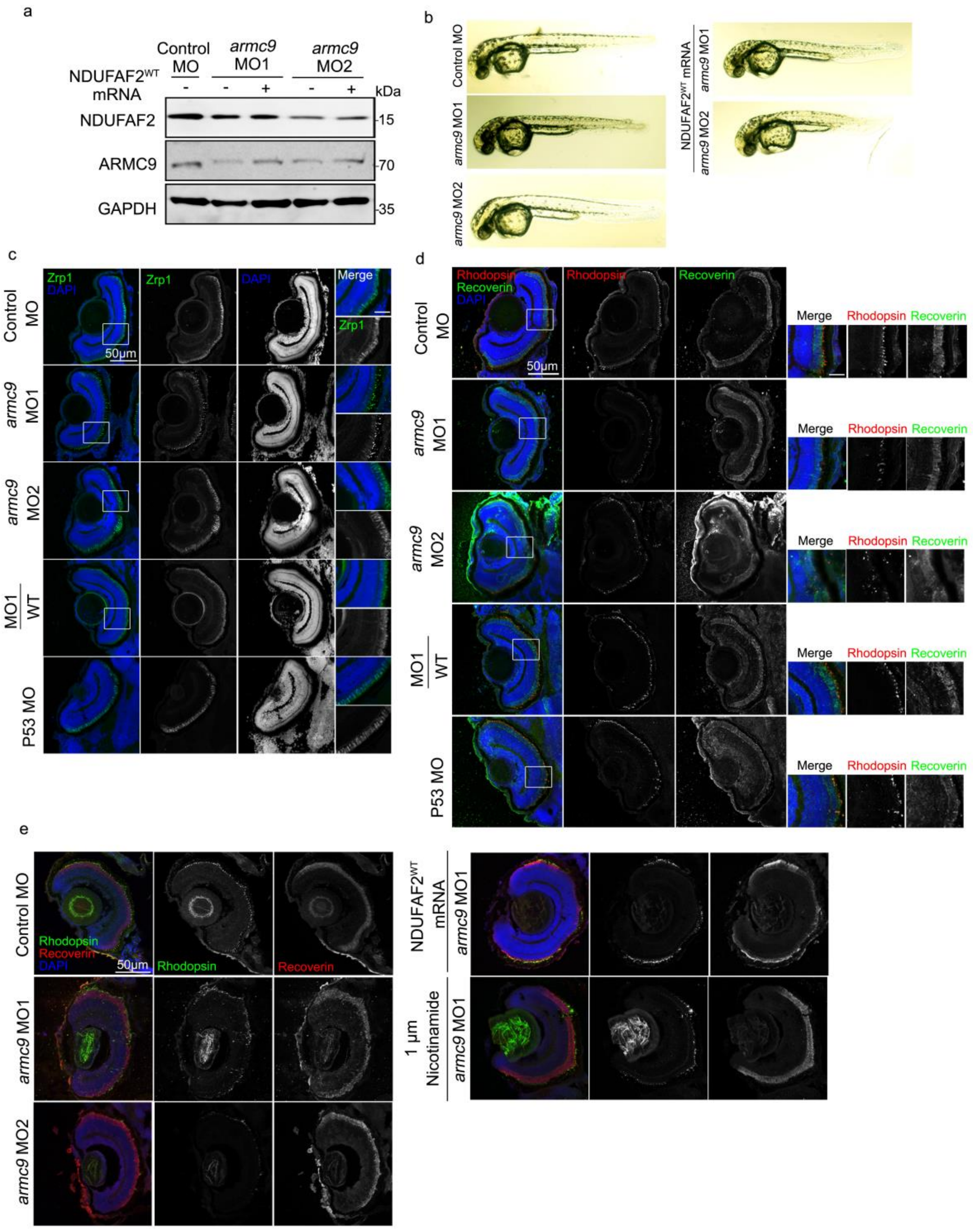

Supplemental Figure 12. NAD+ supplement rescues defective zebrafish photoreceptor in vivo. (a) Protein level of control MO, *armc9* morpholino-injected embryos, Ndufaf2 mRNA and nicotinamide treatment in *armc9* morpholino-injected embryos. Western blot analysis was performed with antibodies against ARMC9, NDUFAF2 and GAPDH. (b) Transmitted light images of body shape in control MO, *p53* and *armc9* morpholino-injected embryos. (c) Images of cone photoreceptors in zebrafish larvae at 5 dpf. Zebrafish larvae stained for Zrp1 (green) and DNA stained

with DAPI (blue). Scale bars as indicated. Scale bar:50 μm. Bars in each graph represent mean ± SD. (d) Images of photoreceptors in zebrafish larvae at 5 dpf. Zebrafish larvae stained for rhodopsin (green) and recoverin (red), DNA stained with DAPI (blue). (e) Images of photoreceptors in zebrafish larvae at 5 dpf with 1 μm of Nicotinamide and NDUFAF2^{WT} re-expressing in ndufaf2 morpholino-injected zebrafish larvae. Zebrafish larvae stained for rhodopsin (green) and recoverin (red), DNA stained with DAPI (blue). Scale bars as indicated. Scale bar (50 μm. Bars in each graph represent mean ± SD.
