# Supplementary figures and images for: Primary cilia formation requires the Leigh syndrome–associated mitochondrial protein NDUFAF2
Source: J Clin Invest. 2024 Jul 1;134(13):e175560. doi: 10.1172/JCI175560 (PMC11213510; doi:10.1172/JCI175560)

Fig.1a

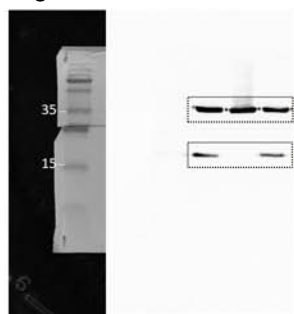

Fig.2a

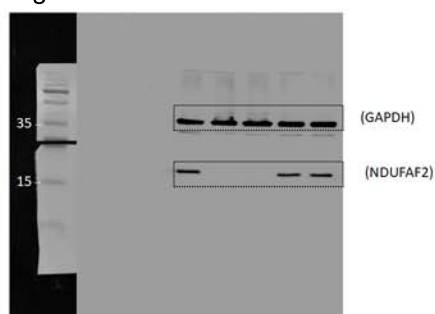

Fig.3a

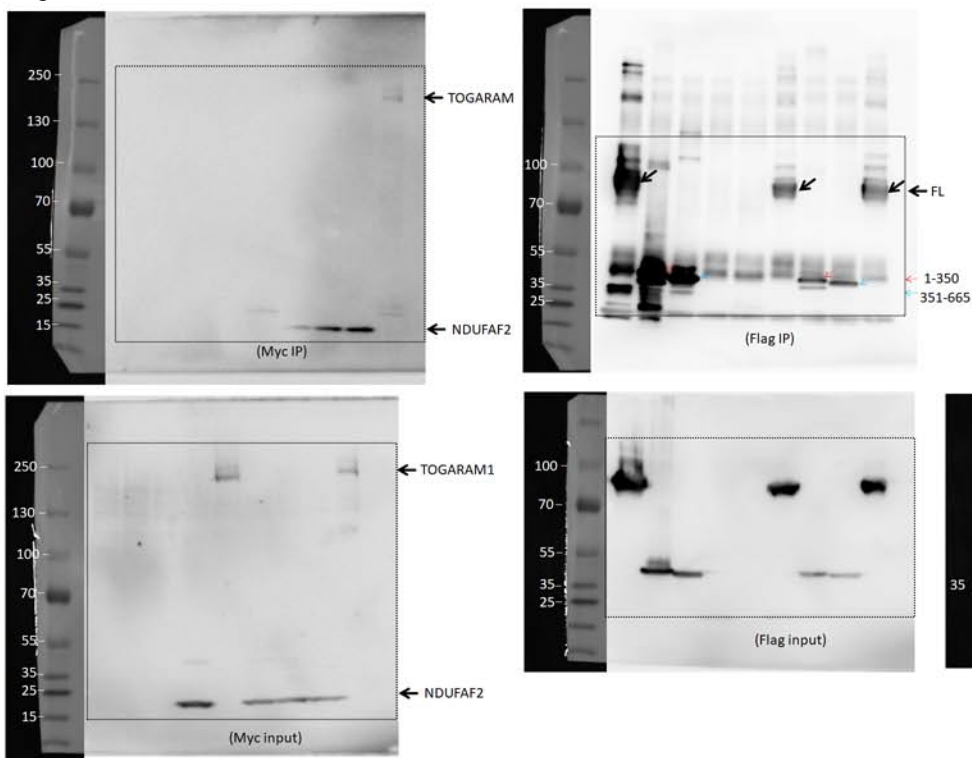

Fig.3b

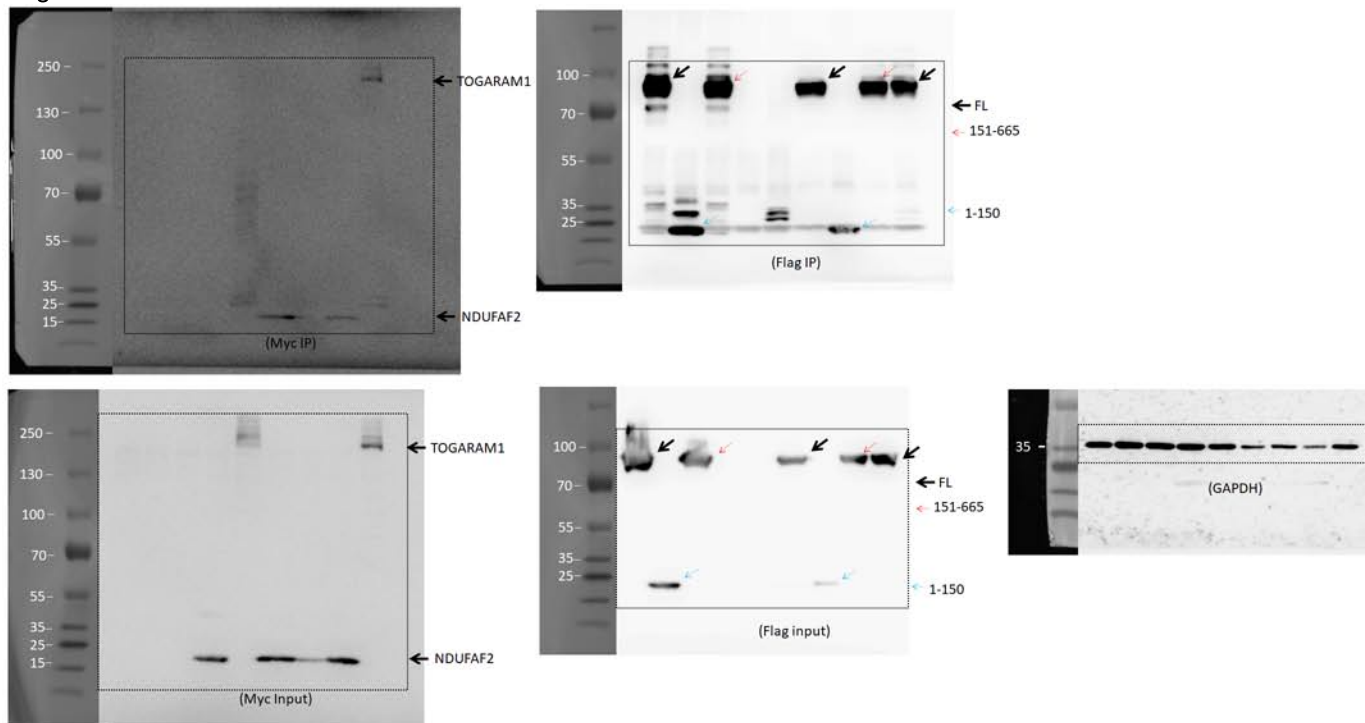

Fig.4b

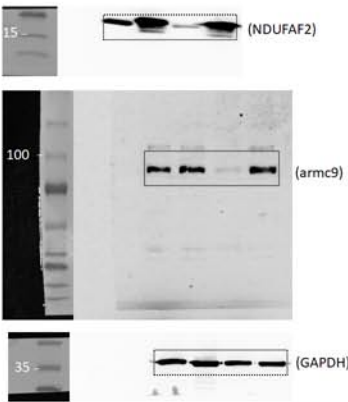

Sup Fig.3e

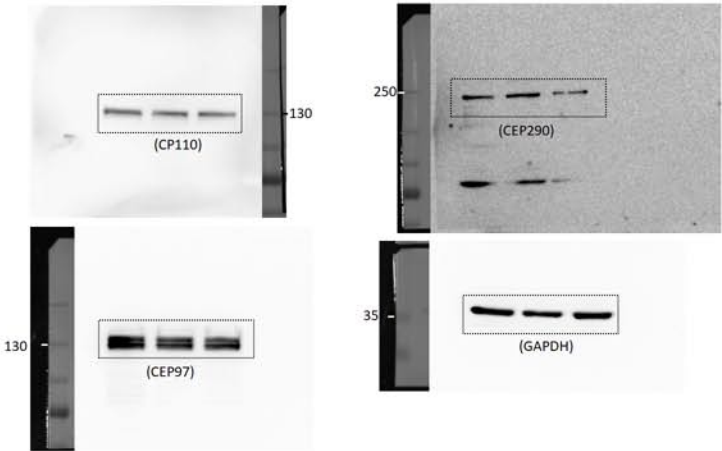

Sup Fig.11a

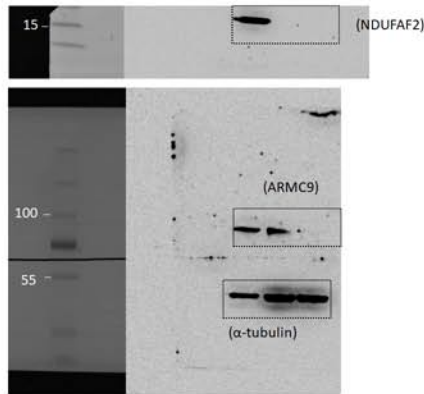

Sup Fig.12a

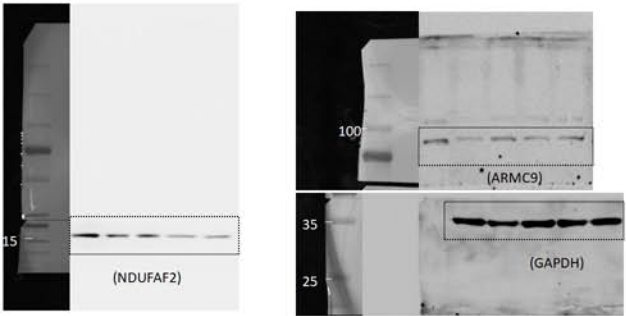

Supplement: Unedited blot and gel images [file jci-134-175560-s177.pdf]
